# Discrepant gut microbiota markers for the classification of obesity-related metabolic abnormalities

**DOI:** 10.1038/s41598-019-49462-w

**Published:** 2019-09-17

**Authors:** Qiang Zeng, Dongfang Li, Yuan He, Yinhu Li, Zhenyu Yang, Xiaolan Zhao, Yanhong Liu, Yu Wang, Jing Sun, Xin Feng, Fei Wang, Jiaxing Chen, Yuejie Zheng, Yonghong Yang, Xuelin Sun, Ximing Xu, Daxi Wang, Toby Kenney, Yiqi Jiang, Hong Gu, Yongli Li, Ke Zhou, Shuaicheng Li, Wenkui Dai

**Affiliations:** 1 Health management institute, People’s Liberation Army General Hospital, Beijing, China; 2 Wuhan National Laboratory for Optoelectronics, Huazhong University of Science and Technology, Wuhan, Hubei Province China; 3 Department of Microbial Research, WeHealthGene Institute, Shenzhen, Guangdong Province China; 4 Joint Laboratory of Micro-ecology and Children’s Health, Shenzhen Children’s Hospital & Shenzhen WeHealthGene Co. Ltd., Shenzhen, Guangdong Province China; 5 National Research Institute for Health, Beijing, China; 6 Department of Computer Science, College of Science and Engineering, City University of Hong Kong, Hong Kong, China; 7 School of Statistics and Data Science, Nankai University, Tianjin, China; 8 Southwest Hospital of Third Military Medical University, Chongqing, China; 9 Health management center, The 910th Hospital of People’s Liberation Army, Quanzhou, Fujian Province China; 10 The China-Japan Union Hospital of Jilin University, Changchun, Jilin Province China; 11 Department of Respiratory, Shenzhen Children’s Hospital, Shenzhen, Guangdong Province China; 12 Department of Cardiology, Longkou People’s Hospital, Longkou, Shandong Province China; 13 Department of Mathematics and Statistics, Dalhousie University, Halifax, Nova Scotia Canada; 14 Department of Health Management, Henan Provincial People’s Hospital, Zhengzhou, Henan Province China

**Keywords:** Data mining, Microbial ecology

## Abstract

The gut microbiota (GM) is related to obesity and other metabolic diseases. To detect GM markers for obesity in patients with different metabolic abnormalities and investigate their relationships with clinical indicators, 1,914 Chinese adults were enrolled for 16S rRNA gene sequencing in this retrospective study. Based on GM composition, Random forest classifiers were constructed to screen the obesity patients with (Group OA) or without metabolic diseases (Group O) from healthy individuals (Group H), and high accuracies were observed for the discrimination of Group O and Group OA (areas under the receiver operating curve (AUC) equal to 0.68 and 0.76, respectively). Furthermore, six GM markers were shared by obesity patients with various metabolic disorders (*Bacteroides*, *Parabacteroides*, *Blautia*, *Alistipes*, *Romboutsia* and *Roseburia*). As for the discrimination with Group O, Group OA exhibited low accuracy (AUC = 0.57). Nonetheless, GM classifications to distinguish between Group O and the obese patients with specific metabolic abnormalities were not accurate (AUC values from 0.59 to 0.66). Common biomarkers were identified for the obesity patients with high uric acid, high serum lipids and high blood pressure, such as *Clostridium XIVa, Bacteroides* and *Roseburia*. A total of 20 genera were associated with multiple significant clinical indicators. For example, *Blautia*, *Romboutsia*, *Ruminococcus2*, *Clostridium sensu stricto* and *Dorea* were positively correlated with indicators of bodyweight (including waistline and body mass index) and serum lipids (including low density lipoprotein, triglyceride and total cholesterol). In contrast, the aforementioned clinical indicators were negatively associated with *Bacteroides*, *Roseburia*, *Butyricicoccus*, *Alistipes, Parasutterella*, *Parabacteroides* and *Clostridium IV*. Generally, these biomarkers hold the potential to predict obesity-related metabolic abnormalities, and interventions based on these biomarkers might be beneficial to weight loss and metabolic risk improvement.

## Introduction

Obesity is an epidemic health issue with a prevalence that reached 39% worldwide according to a survey from the World Health Organization (WHO) in 2016^1^. Prior studies have suggested that obesity increases the risks of other chronic diseases^2–4^. For instance, excessive lipid accumulation in obese patients suppresses insulin signaling^2^, and results in the occurrence of insulin resistance and type 2 diabetes (T2D)^2^. Moreover, adipocyte dysfunction gives rise to systematic inflammation and vascular stiffness, leading to hypertension^5^, chronic kidney diseases (CDK)^3^ and cardiovascular diseases (CVD)^4^.

Increasing evidence has demonstrated that altering gut microbiota (GM) propels the emergence of obesity^6,7^ and correlates with other metabolic disorders in obese patients, such as hypertension^8^, T2D^9^, CDK^10^ and CVD^11^. Through lipopolysaccharides (LPS) derived from bacterial membranes, the GM can trigger inflammatory processes associated with T2D^9^, obesity^12^ and insulin resistance^13^. On the other hand, GM-derived short-chain fatty acids (SCFAs) can enhance insulin sensitivity^14^, affect blood pressure^15^ and stimulate the release of satiety hormones^16^. In addition, GM alterations were identified in different kinds of weight loss, such as calorie restriction^17^, probiotic exposure^18^, drug intervention^19^ and even stomach surgery^20^. Reshaping the GM is effective in weight loss and ameliorating metabolic diseases^6^. However, how the gradual changes in the GM^21^ during weight gain and the onset of metabolic abnormalities in obesity is still unclear.

Emmanuelle Le Chatelier *et al*. have reported GM biomarkers for the early diagnosis of obesity in Europeans^22^, and for obese patients with T2D in other ethnic populations^23^. Moreover, the enrichment of *Enterococcus*, *Blautia*, *Sutterella*, *Klebsiella* and *Collinsella* were found in Chinese obese children and adolescents, as well as the reduction of *Bacteroides*, *Parabacteroides*, *Anaerotruncus* and *Coprobacillus*^24^. Given that GM components are shaped by various factors, including age^25^, diet^26^, ethnicity^27^ and diseases^28^, specific GM biomarkers should be explored for obese adults in China, and biomarker discrepancies need to be described for patients with different metabolic abnormalities.

In this study, 1,914 Chinese adults were enrolled to investigate physiological and GM characteristics in a healthy cohort and an obesity cohort, with or without metabolic abnormalities. We aimed to elucidate: (I) universal GM biomarkers for obese patients, (II) specific GM biomarkers to discriminate obese patients from metabolic abnormalities, and (III) the associations between GM and metabolism relevant indicators. These findings will provide extensive insights into a variety of GM targets for weight loss in obese patients with different clinical symptoms.

## Results

### Characteristics of the cohorts and data output

The recruited 1,914 individuals, who averaged 41 years of age, were from Changchun, Chongqing, Longkou and Quanzhou city, representing four typical lifestyles and living conditions in China (Supplementary Table 1). Of the participants, 58% were male and 11% were healthy individuals with normal body weight and BMI (Supplementary Table 1). The participants were classified into a healthy group (Group H), an obesity group without metabolic abnormalities (Group O) and an obesity group with abnormal clinical indicators (Group OA), depending on their physical examination results and body mass index (BMI) (Table 1). Moreover, Group OA was further classified into 15 subgroups following clinical standards (detailed in Methods, Table 1).

**Table 1:** Summary of group information.

Primary groups	Subgroups	Sample NO.	Clinical feature
Group H	Group H	209	Healthy
Group O	Group O	307	Obesity
Group OA	Group O1	211	Obesity and high UA
	Group O2	289	Obesity and high serum lipid
	Group O3	161	Obesity and high blood pressure
	Group O4	43	Obesity and abnormal renal function
	Group O5	28	Obesity and high serum glucose
	Group O1-2	258	Obesity, high UA and high serum lipid
	Group O1-3	66	Obesity, high UA and high blood pressure
	Group O1-4	39	Obesity, high UA and abnormal renal function
	Group O1-5	8	Obesity, high UA and high serum glucose
	Group O2-3	143	Obesity, high serum lipid and high blood pressure
	Group O2-4	55	Obesity, high serum lipid and abnormal renal function
	Group O2-5	47	Obesity, high serum lipid and high serum glucose
	Group O3-4	18	Obesity, high blood pressure and renal function
	Group O3-5	29	Obesity, high blood pressure and high serum glucose
	Group O4-5	3	Obesity, abnormal renal function and high serum glucose

The sequencing of the 16S rRNA V3-V4 region generated 165,608,482 raw reads with 300 bp paired-end strategy, which were then filtered and connected into 69,726,546 tags for taxonomic identification. After RDP database alignment by DADA2, each sample contained 36,430 ± 19,567 (Mean ± SD) annotated tags, which can be classified into 4.29 ± 0.85 phyla and 29.06 ± 8.49 genera, respectively. In addition, both Group H and Group O exhibited higher genus numbers than those of Group OA (P < 0.001, FDR < 0.001), and the averaged number were 31 ± 9, 32 ± 9 and 28 ± 8 for Group H, Group O and Group OA, respectively (Table 2, Supplementary figure 1). The Shannon index was significantly higher in Group O (1.84 ± 0.60) than in Group H (1.62 ± 0.52, P < 0.001, FDR < 0.001) and Group OA (1.65 ± 0.59) (P < 0.001, FDR < 0.001, Table 2, Supplementary Fig. 1).

**Table 2:** Distribution of genus number and microbial diversity.

	Genus number	Shannon index
Group H	31 ± 9	1.62 ± 0.52
Group O	32 ± 9	1.84 ± 0.60
Group OA	28 ± 8	1.65 ± 0.59
**Comparison on genus number**		
	**P-value**	**FDR**
Group H vs Group O	0.364	0.364
Group H vs Group OA	<0.001	<0.001
Group O vs Group OA	<0.001	<0.001
**Comparison on Shannon index**		
	**P-value**	**FDR**
Group H vs Group O	<0.001	<0.001
Group H vs Group OA	0.445	0.445
Group O vs Group OA	<0.001	<0.001

### Discrepant GM structure identified among Group H, Group O and Group OA

**Figure 1:**
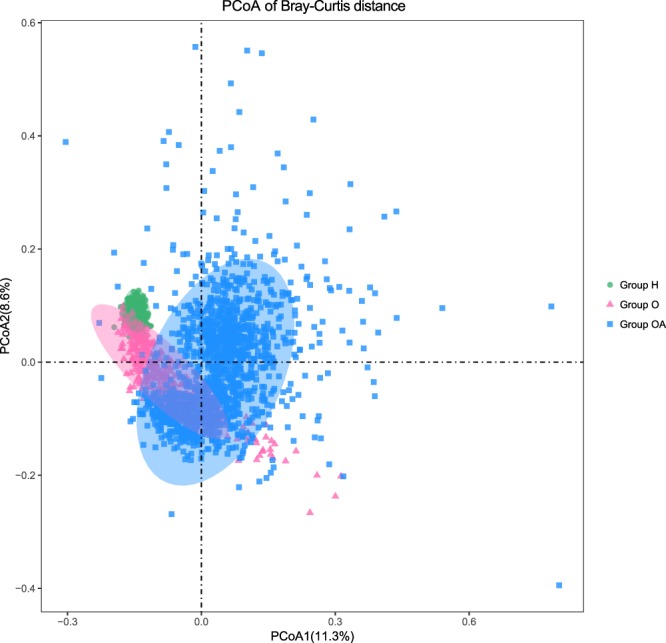
PCoA analysis of Bray-Curtis distance. Green dots, pink triangles and blue squares stand for the samples from Group H, Group O and Group OA, respectively. Ellipses round the geometric represent the standard deviations of the samples.

With principal coordinates analysis (PCoA), we discovered that samples from Group H clustered together, and they were separated from Group OA (Fig. 1). Moreover, samples from Group O partially overlapped with those from Group H and Group OA, while Group OA contained samples with more diversified GM (Fig. 1). Principal component analysis (PCA) was also performed to examine the GM distributions with different metabolic disorders, regions and gender, but no special pattern was observed (Supplementary Fig. 2). In addition, PERMANOVA analysis indicated that the GM composition was significantly associated with geographic region (P = 0.001), BMI (P = 0.001), uric acid (UA, P = 0.001), triglyceride (TG, P = 0.001) and low density lipoprotein (LDL, P = 0.001) (Supplementary Table 2).

### Group OA can be discriminated from Group H with higher accuracy than Group O

**Figure 2:**
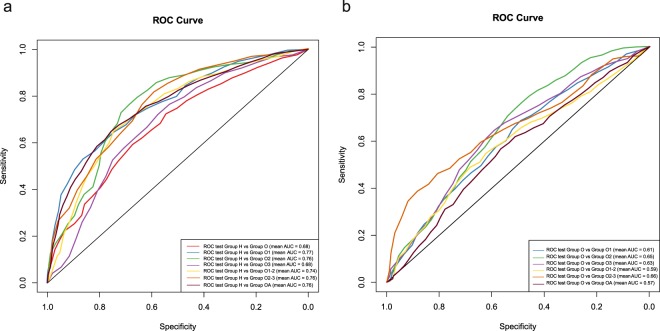
Validation tests of the Random forest classifiers between healthy and obese subjects. (**a)** Cross-validation test was used to detect the accuracy of the biomarkers between healthy and obesity individuals, and their ROC curves were drawn with different colours. **(b)** The accuracy of the biomarkers and Random forest classifiers to discriminate obese patients with different metabolic abnormalities.

Using the Random forest classifier, we identified 13 microbial genus markers discriminating Group O from Group H (Table 3, Supplementary Fig. 3a) with an AUC (area under the receiver operating curve) value equals to 0.68 (Fig. 2a, Table 3). In comparison, the patients from Group OA can be discriminated from the healthy individuals by 47 biomarkers with higher accuracy (AUC = 0.76, Fig. 2a, Table 3). Considering each metabolic abnormality as a separate factor, we detected microbial biomarkers for obese patients with specific metabolic abnormities (Supplementary Fig. 3b–f), and high accuracy were also observed for these obesity subgroups (AUC values from 0.68 to 0.77, Table 3, Fig. 2a). Based on the above Random forest classifiers, 6 common biomarkers were discovered for the obese patients with or without metabolic abnormalities, including *Bacteroides*, *Parabacteroides*, *Blautia*, *Alistipes*, *Romboutsia* and *Roseburia* (Supplementary Fig. 3).

**Table 3:** Assessment of the Random forest classifiers.

Classifier	Biomarker NO.	Accuracy	Sensitivity	Specificity	Precision	F_1_ score	AUC
Group H vs Group O	13	0.65	0.51	0.76	0.60	0.54	0.68
Group H vs Group OA	47	0.70	0.53	0.80	0.62	0.56	0.76
Group H vs Group O1	23	0.68	0.72	0.64	0.67	0.69	0.77
Group H vs Group O2	17	0.73	0.66	0.79	0.71	0.67	0.76
Group H vs Group O3	11	0.62	0.74	0.46	0.65	0.69	0.68
Group H vs Group O1-2	10	0.70	0.65	0.74	0.67	0.65	0.74
Group H vs Group O2-3	20	0.72	0.80	0.58	0.77	0.77	0.76
Group O vs Group OA	24	0.51	0.45	0.57	0.48	0.46	0.57
Group O vs Group O1	44	0.59	0.75	0.35	0.63	0.68	0.61
Group O vs Group O2	15	0.61	0.61	0.61	0.62	0.61	0.65
Group O vs Group O3	19	0.64	0.80	0.32	0.70	0.74	0.63
Group O vs Group O1-2	19	0.56	0.64	0.46	0.59	0.61	0.59
Group O vs Group O2-3	42	0.71	0.90	0.27	0.74	0.81	0.66

### GM biomarkers to differentiate Group OA from Group O

In total, 24 GM biomarkers were discovered to discriminate Group OA from Group O (Table 3) with AUC equals to 0.57 (Fig. 2b, Table 3). To understand the GM characteristics in obese individuals with specific metabolic abnormalities, GM biomarkers were detected among subgroups in Group OA and Group O (Supplementary Fig. 4b–e), and their AUC values ranged from 0.59 to 0.66 (Fig. 2b, Table 3), which were lower than those between obese patients and healthy individuals (AUC values from 0.68 to 0.77, Fig. 2a, Table 3). Despite of diversified metabolic abnormalities, *Clostridium XIVa, Bacteroides*, and *Roseburia* were discovered for the classification of Group O1, Group O2, and Group O3 (Supplementary Fig. 4). Moreover, *Gemmiger*, *Dorea*, *Faecalibacterium*, *Blautia* and *Coprococcus* were GM biomarkers that shared in obese patients with different metabolic abnormalities (Supplementary Fig. 4).

### GM biomarkers are correlated with multiple clinical indicators that are also involved in complex relationships

**Figure 3:**
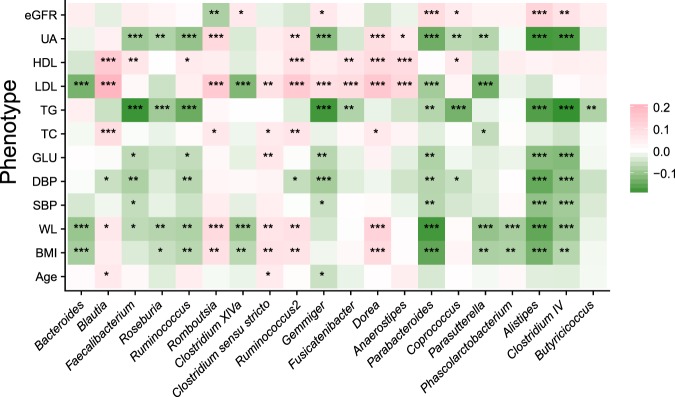
Relationships between GM components and clinical indicators. A Spearman correlation analysis was executed between GM components and clinical indicators. A total of 20 genera were selected, and each genus was significantly correlated with more than one phenotype. Red and green colour indicate positive and negative relationships, respectively. FDR-adjusted P values were indicated by asterisks (one, two and three asterisks indicate P values smaller than 0.05, 0.01 and 0.001, respectively).

A total of 20 microbial genera were associated with multiple significant clinical indicators (Fig. 3, Supplementary Table 3). As a dominant genus, *Bacteroides* was negatively correlated with LDL (r = −0.13, P < 0.001, FDR < 0.001), waistline (WL, r = −0.10, P < 0.001, FDR < 0.001) and BMI (r = −0.09, P < 0.001, FDR = 0.001). Meanwhile, *Roseburia, Parabacteroides, Parasutterella, Alistipes*, *Clostridium IV* and *Butyricicoccus* were negatively correlated with a variety of clinical indicators, including body weight (including BMI and WL), serum lipids (including LDL, TG and total cholesterol (TC)), blood pressure (including systolic blood pressure (SBP) and diastolic blood pressure (DBP)), blood glucose (GLU) and uric acid (UA) (Fig. 3, Supplementary Table 3). Conversely, *Blautia* was positively correlated with LDL (r = 0.20, P < 0.001, FDR < 0.001), TC (r = 0.09, P < 0.001, FDR < 0.001), and WL (r = 0.06, P = 0.005, FDR = 0.014) (Fig. 3, Supplementary Table 3). Moreover, *Romboutsia*, *Ruminococcus2*, *Clostridium sensu stricto* and *Dorea* were positively and significantly associated with body weight, serum lipids and UA (P < 0.05, FDR < 0.05, Fig. 3, Supplementary Table 3).

**Figure 4:**
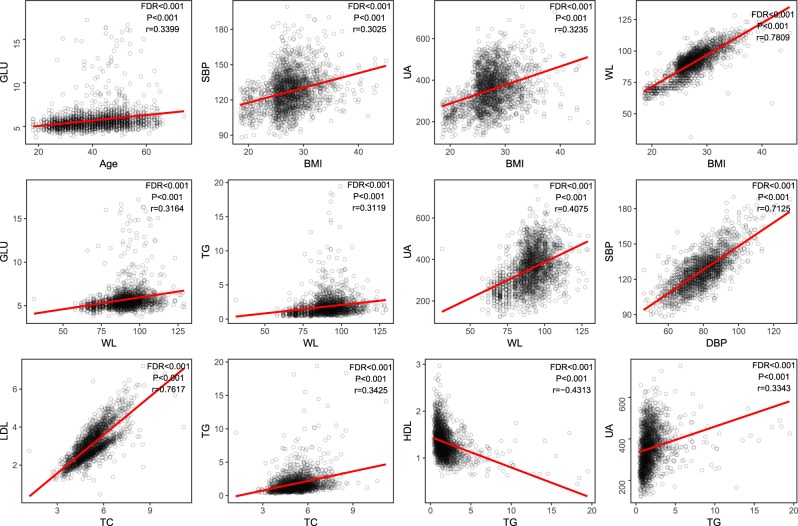
Associations among different clinical indicators. The relationships among different phenotypes were suggested by Spearman correlation coefficients. The correlations were kept when the coefficients were larger than 0.3 or smaller than −0.3 (P < 0.001, FDR < 0.05), and the coefficients of linear regression were suggested by the red lines in the pictures.

A positive association between WL and BMI (r = 0.78, P < 0.001, FDR < 0.001, Fig. 4) was also identified in Chinese adults, and the levels of SBP (r = 0.30, P < 0.001, FDR < 0.001) and UA (r = 0.32, P < 0.001, FDR < 0.001) augmented BMI (Fig. 4). WL was positively correlated with GLU (r = 0.32, P < 0.001, FDR < 0.001), TG (r = 0.31, P < 0.001, FDR < 0.001) and UA (r = 0.41, P < 0.001, FDR < 0.001), which are potential indicators for diabetes, hyperlipaemia and hyperuricaemia, respectively (Fig. 4). Furthermore, we discovered a positive association between UA and TG (r = 0.33, P < 0.001, FDR < 0.001, Fig. 4).

## Discussion

In this retrospective study, we detected the GM characters of obese patients with various metabolic abnormalities. Although studies have revealed the decreased bacterial diversity in obese patients^29,30^, in current study higher bacterial diversity was detected in obese patients without metabolic abnormalities than in healthy individuals. Therefore, we hypothesized that specific bacteria and their associations with obesity should be understood, other than bacterial diversity which might be affected by diet, body size and other factors^31^. With the onset of metabolic abnormalities in obese adults, aggravated GM dysbiosis brings about dwindling bacterial diversity and genus number^29^. Moreover, obvious inter-group GM discrepancy was observed between Group H and Group OA after PCoA analysis, while the Group O seemed to be the intermediate state of healthy and obese with metabolic abnormalities. We therefore suggest that gradual GM changes occurred with the aggravation of obesity and the occurrence of other metabolic diseases.

To differentiate obese patients from healthy individuals, six universal biomarkers were identified through random forest classifiers, including *Bacteroides*, *Parabacteroides*, *Blautia*, *Alistipes*, *Romboutsia* and *Roseburia*. Interestingly, most of those genera have been found to interact with host immune system. For example, *Bacteroides* has been revealed to promote the differentiation of regulatory T cells (Treg) and protect against inflammatory reactions^32^. Meanwhile, systemic inflammatory responses can be suppressed by *Parabacteroides* through its regulations of IL-10 and Treg cells^33^. Conversely, *Alistipes* would trigger inflammatory reactions in hosts, and the genus was also found abundant in Chinese T2D patients^9^. Based on their close relationships with host immune system, these biomarkers can be applied for the early diagnosis of obesity and other metabolic risks, given the observed high accuracy (AUC ranged from 0.68 to 0.77). Furthermore, these biomarkers seem to be population specific. For a Danish population^22^, 18 biomarkers have been identified to differentiate obese and lean individuals, including species from *Bacteroides, Clostridium, Faecalibacterium* and *Ruminococcus*. However, only one biomarker was commonly found in our Chinese cohort. On the other hand, nine obese-associated genera were reported in Chinese children^24^, and three of them were consistent with the findings in this study, including *Bacteroides*, *Parabacteroides* and *Blautia*. These outcomes enlightened us that specific GM interventions should be considered for different populations with various lifestyles^27^.

Compared to the obese patients without abnormalities, the patients with metabolic abnormalities demonstrated altered GM components, and *Clostridium XIVa* contributed to the discrimination of obese patients with high UA, serum lipid or blood pressure. A previous report documented that *Clostridium XIVa* could produce butyrate^34^, and it would suppress systemic inflammatory responses. In addition, *Roseburia* was also applied for the differentiation of obese patients with high UA, serum lipid or blood pressure. As a butyrate-producing bacterium^35^, *Roseburia* could stimulate the differentiation of Treg cells, which was beneficial for the alleviation of inflammation. Despite of distinct clinical symptoms, the obese patients with different metabolic abnormalities shared some GM biomarker, such as *Blautia*, *Dorea* and *Gemmiger*. As an acetate producer^36^, *Blautia* can drive insulin release and promote metabolic syndromes, such as hypertriglyceridaemia, fatty liver disease and insulin resistance^16^. Meanwhile, *Dorea* was negatively associated with insulin resistance^37^, and *Gemmiger* would aggregate inflammatory reactions in the hosts through its colonization factors^38^. These biomarkers indicated the common GM alterations in obese patients with different metabolic abnormalities, so other factors (such as genetic variation) might involve in the occurrence of the different metabolic diseases^39^. Based on such observations, we also speculated that obesity-related GM alterations laid the foundation for the occurrence of metabolic disorders, and other specific pathogenic perspectives need to be explored beyond the GM dysbiosis.

The associations between bacterial components and the clinical indicators were explored. Since *Faecalibacterium* and *Butyricicoccus* could secret butyrate^40^ and boost insulin sensitivity^41^, their negative correlations with LDL, GLU, UA, TC and BMI were discovered. In contrast, *Blautia* was positively correlated with the aforementioned clinical indicators due to acetate secretion^36^. Given that *Faecalibacterium* and *Butyricicoccus* play opposite roles as compared with *Blautia*, we speculated that synergism and antagonism inside the microbial community were also crucial for obesity development. In addition, *Parabacteroides*^33^ and *Clostridium IV*^42^ could suppress inflammatory responses, and they were negatively associated with the blood pressure, blood lipid and GLU. Since some of the aforementioned bacteria were GM biomarkers in the obese patients, we deduced that these bacteria might be the potential targets for the interference of metabolic disorders, and the corresponding clinical symptoms would possibly be relieved based on these host-microbial relationships. In addition, the relationships among physiological parameters suggested that fat primarily accumulated at the waist in Chinese populations when obesity occurred^43^, and increased waistline was positively associated with elevated blood pressure, blood sugar, UA and TG. Hence, waistline can be recognized as a signal for the occurrence of metabolic abnormalities in Chinese adults.

A limitation of the current research is that the validation accuracy of the biomarkers was not testified in different populations. Since GM composition was affected by ethnicity and lifestyles^27^, the obesity cohorts from other populations would benefit to understand the application scope of the biomarkers. In further study, addition work is also imperative: I) examine the genetic characters in patients with different metabolic diseases; II) perform metagenomic sequencing to evaluate the microbial functions; III) explore the alteration of intestinal metabolites in patients with metabolic diseases, and their associations with gut microbiome.

In conclusion, the study detected the GM features in the Chinese obese adults with large cohort, furnished genus markers for obese patients with different metabolic abnormalities, and illustrated the associations between bacterial commensals and various clinical indicators. These findings suggested the roles of GM in the pathogenesis of metabolic diseases, and offered potential GM targets for the adjuvant interventions on the treatment of obesity with metabolic abnormalities.

## Methods

### Ethics statement

This study was approved by the Ethics Committee of The General Hospital of the People’s Liberation Army (PLAGH) under registration number S2016-068-01, and the research was carried out according to The Code of Ethics of the World Medical Association. All participants provided signed informed consents, and volunteered to be investigated for scientific research.

### Participant recruitment

Randomized volunteers were recruited in four hospitals in China: The 180th Hospital of People’s Liberation Army of China (Quanzhou, China), China-Japan Union Hospital (Changchun, China), Southwest Hospital (Chongqing, China) and Longkou People’s Hospital (Longkou, China). A total of 2,058 Han Chinese joined the study, and they completed physical testing including height, weight, waistline and blood pressure. By using a blood auto-analyzer (Beckman Coulter AU5800, Brea, CA, USA), blood testing was carried out in the participants to evaluate the health condition consist of GLU, TC, TG, LDL, high density lipoprotein (HDL), UA and eGFR (Supplementary Table 1).

The participants who satisfied the following criteria were excluded from this study: (I) younger than 18 years or older than 75 years; (II) exposed to antibiotic, probiotics or proton pump inhibitor 1 month before physical examination; (III) suffered from diarrhoea, constipation, haematochezia or other gastrointestinal infectious diseases 1 month prior to physical examination; (IV) experienced enema or other gastroenterology operations 1 month before physical examination; (V) suffered from mental disorders (*e.g*., depression, anxiety and post-traumatic stress), autoimmune diseases (*e.g*. type 1 diabetes, rheumatoid arthritis, multiple sclerosis and psoriasis.) or hereditary diseases (*e.g*., thalassemia, hereditary deafness and phenylketonuria); (VI) had drug abuse history; (VII) exposed to antibiotic, probiotic, or proton pump inhibitors 4 weeks prior to the study. Finally, 1,914 individuals, from whom faecal samples were collected, were enrolled in the study between Jan. 2016 and Sep. 2016.

### Grouping based on clinical indicators

The participants were first divided into 2 groups: a healthy group and an obesity group. The healthy group (Group H) included individuals who passed their physical examinations with a normal BMI (between 18.5 and 23.99)^44^. On the other hand, overweight and obese patients, whose BMI was larger than 24, were assigned to the obesity group in this study. Using published previously clinical standards, five kinds of metabolic abnormalities were defined in the obesity cohorts, including high UA^45^ (>416 µmol/L in male or >350 µmol/L in female), high serum lipid^46^ (TC ≥ 6.22 mmol/L, TG ≥ 2.26 mmol/L, LDL ≥ 4.14 mmol/L and/or HDL < 1.04 mmol/L), high blood pressure^47^ (SBP ≥ 140 mmHg, DBP ≥ 90 mmHg), abnormal renal function^48^ (eGFR < 60 ml/Min/Hight^2^) and high serum glucose^49^ (≥7.0 mmol/L). Relying on clinical indicators and personal confirmation, the obese patients were divided into obesity groups with (Group OA) or without metabolic abnormalities (Group O), and then Group OA was subdivided into 15 obesity groups with different metabolic abnormalities (Table 1). To avoid data deviation, groups with less than 100 individuals were removed from subsequent analysis.

### Faecal sample collection

The sterile stool collection tubes (Axygen, California, USA) were delivered to the participants, and fresh stools were collected from them when they underwent physical examination. Two kinds of tools were prepared to collect different types of stool: I) a swab (Huachenyang Technology CO., LTD, Shenzhen, China) was used to collect hard stools, and approximately 5 grams of stools was obtained from each person; II) a dropper (Shanghai Truelab Lab, Shanghai, China) was applied to collect loose stools, and approximately 5 ml of stools was acquired from each person. The stool samples were preserved in stool collection tubes, and then transferred to a −80 °C refrigerator for long-term storage within half an hour. Contamination from urine or the environment was avoided during stool sample collection.

### DNA extraction, library construction and sequencing

Microbial DNA was extracted from stool samples using a Power Soil DNA Isolation Kit (Mo Bio Laboratories, Carlsbad, USA). The V3-V4 region of the 16S rRNA gene was amplified by primers 338F and 806R using a PCR kit (TransGenAP221-02, Peking, China). The quality of the PCR products was detected by Qubit (Thermo Fisher, Singapore), and the qualified DNA was prepared for library construction (TruSeq DNA PCR-Free kit, Illumina, San Diego, USA). The libraries were sequenced on an Illumina Miseq sequencing platform (Illumina, San Diego, USA) with 300 base pairs.

### Data filtering and taxonomical annotation

Raw sequenced reads were first paired-filtered for adapter contamination (>15 bases), low quality (10 bases with <Q20), and N contained (>1 base) using a self-programmed script. Then, the filtered reads were processed with the DADA2 (v1.6.0) package^50^ in R (v3.4.4). Bases were trimmed from the reads if their quality scores were lower than 2, and the trimmed reads were discarded if their lengths were shorter than 200 bps. Then, the sequence variants were inferred for each sample with default parameters and merged into tags. After chimeras removal, qualified tags were aligned to the RDP 16S rRNA database (trainset 16/release 11.5)^51^ to obtain corresponding taxonomic profiling. The Shannon index was calculated to evaluate samples biodiversity by using the vegan package in R.

### PCoA and PCA analysis

Based on the genus profiling, Bray-Curits distances among samples were calculated by using package “vegdist” in R. Then, the Bray-Curits matrix was used for PCoA analysis by using “pcoa” in R (Supplementary scripts). With genus profiling, PCA was performed using package “ade4” in R. The PCoA and PCA results were plotted with the package ggplot2 in R.

### PERMANOVA to evaluate the influence of physical indices

With the GM composition of all samples, PERMANOVA^52^ (Permutational Multivariate Analysis of Variance) was carried out to assess the impacts of various physical indices, which are listed in Supplementary Table 2. Based on the Euclidean distances among the samples, the environmental factors which affect GM compositions significantly were detected by using the vegan package in R with 9,999 permutations. The related script was contained in Supplementary scripts.

### Associations between GM composition and clinical indicators

The genera were screened if their abundances lower than 0.05% in more than 50% of samples (Supplementary Table 3), and Spearman correlation analysis was executed between the filtered genera and clinical indicators with all samples (using “cor” in R). Meanwhile, the relationships among different clinical indicators were also analyzed with Spearman correlation, and the significant relationships (r > 0.3, P < 0.05) were kept for further study.

### Construction of random forest models and selection of GM markers

With the relative abundances of genera, random forest classifiers^53^ were constructed using a three-step scheme using package randomForest in R. Firstly, the samples in each group were randomized into 2 sets: a discovery data set (70% of the samples) and a validation data set (30% of the samples). Secondly, random forest models were constructed by the discovery data sets comprising the two compared groups. Finally, the constructed models were applied to the validation data sets comprising the compared groups, and compared with the actual category of the samples. The model validity was evaluated with precision, sensitivity, specificity, precision, F1 score and AUC value with 10 repeats, and the ROC curves were plotted using the R package “pROC”. The detailed script and parameters were shown in Supplementary scripts.

GM biomarkers were obtained from the constructed random forest models. Based on the optimal branch number and Gini values, genera were selected as candidate biomarkers. Since the models were constructed with 10 repeats, candidate biomarkers that arose over 8 times among 10 repeats were selected as final GM biomarkers for discrimination of the two compared groups.

### Statistics

All statistical analyses were performed in R (version 3.4.1). Wilcoxon rank-sum test was executed on Shannon index and genus number between different obese groups by using “Wilcox.test” in R, and the statistical difference was examined among Group H, Group O and Group OA using “kruskal.test” or “chisq.test” in R. Spearman correlation was used to evaluate the associations between GM and clinical indicators, and the relationships among different clinical indicators (using “cor” in R). Statistical results from the previous tests were adjusted with Benjamini-Hochberg method (FDR < 0.05) using “p.adjust”, and were plotted using the package “ggplot2” in R.

## Supplementary information

Supplementary files

## Data Availability

The DNA sequencing data is available in NCBI Sequence Read Archive (SRA) under the Accession Number SRP125854.
