# Supplementary material for: Discrepant gut microbiota markers for the classification of obesity-related metabolic abnormalities
Source: Sci Rep. 2019 Sep 17;9:13424. doi: 10.1038/s41598-019-49462-w (PMC6748942; doi:10.1038/s41598-019-49462-w)
Supplement: Supplementary file 1 — Supplementary files [file 41598_2019_49462_MOESM1_ESM.docx]

**Discrepant gut microbiota markers for the classification of obesity-related metabolic abnormalities**

Qiang Zeng^1,†^, Dongfang Li^2,3,4,†^, Yuan He^5,†^, Yinhu Li^6,†^, Zhenyu Yang^7,†^, Xiaolan Zhao^8^, Yanhong Liu^,3,4^, Yu Wang^9^, Jing Sun^10^, Xin Feng^3, 4^, Fei Wang^1^, Jiaxing Chen^6^, Yuejie Zheng^4,11^, Yonghong Yang^4,11^, Xuelin Sun^12^, Ximing Xu^7,^, Daxi Wang^3,4^, Toby Kenney^13^, Yiqi Jiang^6^, Hong Gu^13^, Yongli Li^14^, Ke Zhou^2,‡^, Shuaicheng Li^6,‡^, Wenkui Dai^6,‡^

1. Health management institute, People's Liberation Army General Hospital, Beijing, China
2. Wuhan National Laboratory for Optoelectronics, Huazhong University of Science and Technology, Wuhan, Hubei Province, China
3. Department of Microbial Research, WeHealthGene Institute, Shenzhen, Guangdong Province, China
4. Joint Laboratory of Micro-ecology and Children’s Health, Shenzhen Children’s Hospital & Shenzhen WeHealthGene Co. Ltd., Shenzhen, Guangdong Province, China
5. National Research Institute for Health, Beijing, China
6. Department of Computer Science, College of Science and Engineering, City University of Hong Kong, Hong Kong, China
7. School of Statistics and Data Science, Nankai University, Tianjin, China
8. Southwest Hospital of Third Military Medical University, Chongqing, China
9. Health management center, The 910th Hospital of People's Liberation Army, Quanzhou, Fujian Province, China
10. The China-Japan Union Hospital of Jilin University, Changchun, Jilin Province, China
11. Department of Respiratory, Shenzhen Children’s Hospital, Shenzhen, Guangdong Province, China
12. Department of Cardiology, Longkou People's Hospital, Longkou, Shandong Province, China
13. Department of Mathematics and Statistics, Dalhousie University, Halifax, Nova Scotia, Canada
14. Department of Health Management, Henan Provincial People’s Hospital, Zhengzhou, Henan Province, China

^†^**These authors contributed equally to this work.**

^‡^**Corresponding authors**

Ke Zhou; Address: Wuhan National Laboratory for Optoelectronics, Huazhong University of Science and Technology, Wuhan 430074, China; Telephone: 0086-027-87559790; Fax: 0086-027-87559790; Email: [k.zhou@hust.edu.cn](mailto:k.zhou@hust.edu.cn).

Shuaicheng Li; Address: Department of Computer Science, College of Science and Engineering, City University of Hong Kong, Kowloon, Hong Kong, 999077, China; Telephone: 0852-3442-9412; Fax: 0852-3442-0503; Email: [shuaicli@cityu.edu.hk](mailto:shuaicli@cityu.edu.hk).

Wenkui Dai; Address: Department of Computer Science, College of Science and Engineering, City University of Hong Kong, Kowloon, Hong Kong, 999077, China; Telephone: 0852-3442-9412; Fax: 0852-3442-0503; Email: [wenkuidai2-c@my.cityu.edu.hk](mailto:wenkuidai2-c@my.cityu.edu.hk).

**
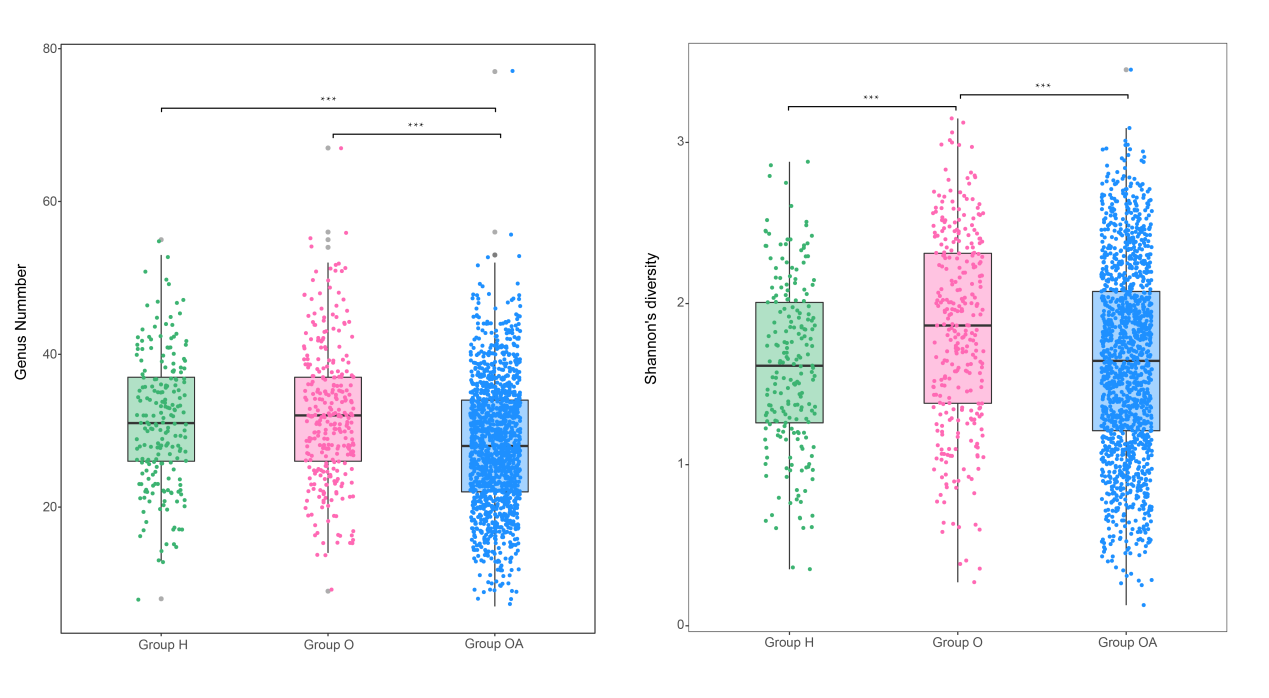
Supplementary figure 1. Distribution of genus number and microbial diversity.** The number of genera was counted for each sample, and microbial diversity was suggested by the Shannon index. The Wilcoxon rank-sum test was applied to detect differences among every pair of groups. FDR-adjusted P values are indicated by asterisks (one, two and three asterisks indicate P values smaller than 0.05, 0.01 and 0.001, respectively).

**Supplementary figure 2. PCA distribution in 1,914 microbial samples.** GM distribution was mainly affected by *Bacteroides*, *Blautia*, *Prevotella*, *Ruminococcus* and *Clostridium sensu stricto*. In the left picture, groups with over 100 samples are marked with different colours, and other groups are labeled as “other”. In the right picture, samples from four regions are marked by different colours, while different shapes indicate the feature of gender.

**
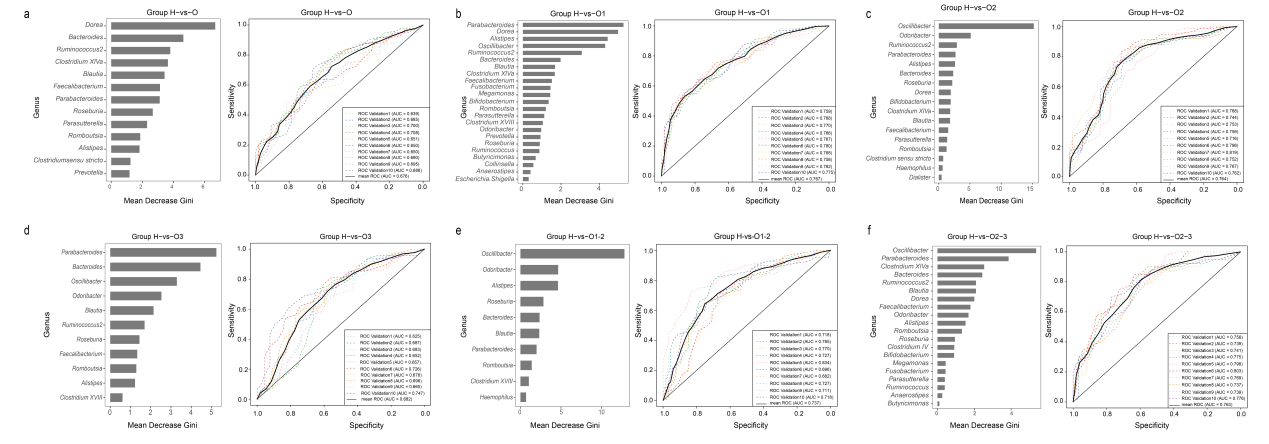
**

**Supplementary figure 3. GM biomarkers and their validation accuracy for the discrimination of obesity cohorts.** Following optimal variation numbers indicated by random forest classifiers, GM biomarkers that emerged over 8 times among 10 repeats were selected for Group O (a), Group O1 (b), Group O2 (c), Group O3 (d), Group O1-2 (e) and Group O2-3 (f). The averaged Gini values were applied to indicate the contributions of biomarkers to the classification. In addition, the accuracy of biomarkers was tested by using validation sets, and their AUC values were calculated. The ROC curves were drawn with ten repeats in different colours.


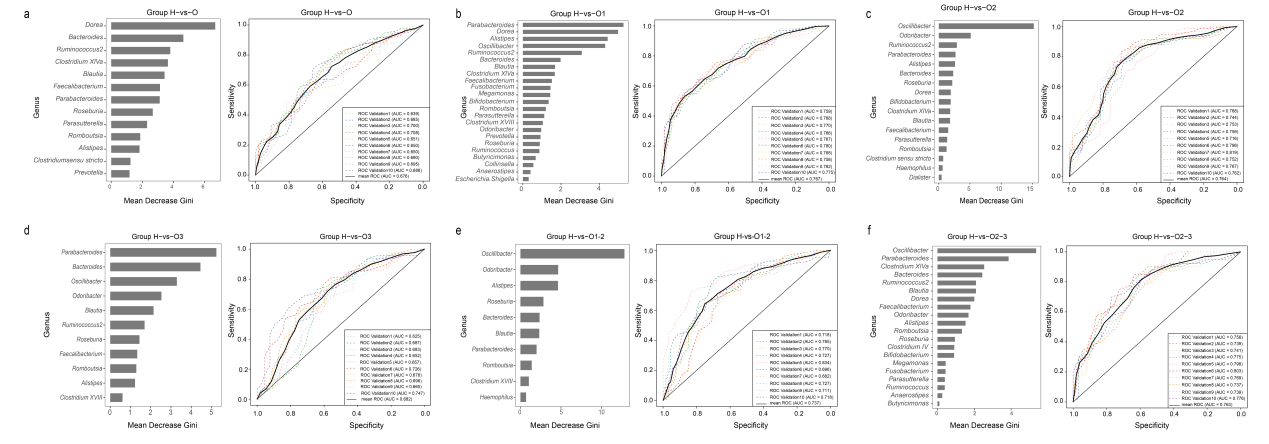


**Supplementary figure 4. GM biomarkers and their accuracy in differentiating Group O from Group OA.** Compared with Group O, genus markers were explored for Group O1 (a), Group O2 (b), Group O3 (c), Group O1-2 (d) and Group O2-3 (e). Biomarker accuracy was evaluated by AUC values, and ROC curves were drawn along with ten repeats in different colours. The AUC values ranged from 0.59 to 0.66.

**Supplementary table 1. Background information of the samples**

| **Characteristics** | **Group H** | **Group O** | **Group OA** | **P-value** |
| --- | --- | --- | --- | --- |
| **Sample number** | 209 | 307 | 1398 |  |
| **Age** (year) | 32.641±7.978 | 39.547±9.828 | 42.282±10.290 | <0.001^#^ |
| **Gender** (male) | 18 | 133 | 963 | <0.001^##^ |
| **BMI** (kg/m2)* | 21.161±1.544 | 27.291±2.401 | 28.536±3.156 | <0.001^#^ |
| **WL** (cm)* | 71.292±6.250 | 89.071±10.383 | 94.643±36.445 | <0.001^#^ |
| **SBP** (mmHg)* | 116.536±10.540 | 119.870±11.331 | 130.346±15.750 | <0.001^#^ |
| **DBP** (mmHg)* | 71.268±9.120 | 73.254±9.466 | 81.522±11.269 | <0.001^#^ |
| **GLU** (mmol/L)* | 4.985±0.423 | 5.361±0.564 | 5.871±1.601 | <0.001^#^ |
| **Blood lipid** | | | | |
| **TC** (mmol/L)* | 4.610±0.729 | 4.933±0.676 | 5.429±1.015 | <0.001^#^ |
| **TG** (mmol/L)* | 0.927±0.374 | 1.177±0.446 | 2.113±1.721 | <0.001^#^ |
| **LDL** (mmol/L)* | 2.611±0.607 | 2.876±0.638 | 3.214±0.908 | <0.001^#^ |
| **HDL** (mmol/L)* | 1.417±0.256 | 1.452±0.282 | 1.391±3.669 | <0.001^#^ |
| **UA** (μmol/L)* | 253.564±51.959 | 299.784±63.465 | 381.727±90.123 | <0.001^#^ |
| **eGFR** (mL/Min/Height^2^)* | 141.847±21.649 | 143.713±35.424 | 124.246±33.937 | <0.001^#^ |
| **Area** | | | | |
| ChangChun | 175 | 36 | 88 | <0.001^#^ |
| ChongQing | 16 | 81 | 612 |  |
| LongKou | 0 | 23 | 94 |  |
| QuanZhou | 18 | 167 | 604 |  |

***BMI:** Body mass index; **WL:** Waistline; **SBP:** Systolic blood pressure; **DBP:** Diastolic blood pressure; **GLU:** Blood glucose; **TC:** Total cholesterol; **TG:** Triglyceride; **LDL:** Low density lipoprotein; **HDL:** High density lipoprotein; **UA:** Uric acid; **eGFR:** epidermal growth factor receptor.

**^#^:** Kruskal-Wallis test.

**^##^:** Chi-square test.

**Supplementary table 2. PERMANOVA ranking associations between GM and clinical indicators**

| **Phenotype** | **Degree of freedom** | **R2** | **P-value** | |
| --- | --- | --- | --- | --- |
| **Age** | 1 | 0.00187 | 0.005 | ** |
| **BMI*** | 1 | 0.00536 | 0.001 | *** |
| **WL*** | 1 | 0.00027 | 0.823 |  |
| **SBP*** | 1 | 0.00131 | 0.018 | * |
| **DBP*** | 1 | 0.00191 | 0.005 | ** |
| **GLU*** | 1 | 0.0004 | 0.489 |  |
| **TC*** | 1 | 0.00153 | 0.014 | * |
| **TG*** | 1 | 0.00461 | 0.001 | *** |
| **LDL*** | 1 | 0.02001 | 0.001 | *** |
| **HDL*** | 1 | 0.00059 | 0.313 |  |
| **UA*** | 1 | 0.00307 | 0.001 | *** |
| **eGFR*** | 1 | 0.00108 | 0.041 | * |
| **Region** | 3 | 0.03147 | 0.001 | *** |

***BMI:** Body mass index; **WL:** Waistline; **SBP:** Systolic blood pressure; **DBP:** Diastolic blood pressure; **GLU:** Blood glucose; **TC:** Total cholesterol; **TG:** Triglyceride; **LDL:** Low density lipoprotein; **HDL:** High density lipoprotein; **UA:** Uric acid; **eGFR:** epidermal growth factor receptor.

**Supplementary table 3. Spearman correlation coefficients between GM components and clinical indicators (R value)**

| **Genus** | **Age** | **BMI*** | **WL*** | **SBP*** | **DBP*** | **GLU*** | **TC*** | **TG*** | **LDL*** | **HDL*** | **UA*** | **eGFR*** |
| --- | --- | --- | --- | --- | --- | --- | --- | --- | --- | --- | --- | --- |
| *Abiotrophia* | -0.019263 | 0.009343 | 0.016575 | -0.00009 | -0.012097 | -0.016326 | -0.034475 | -0.029821 | -0.009174 | 0.009637 | 0.018422 | 0.027837 |
| *Acetanaerobacterium* | 0.002436 | -0.061938 | -0.062143 | -0.047745 | -0.04661 | -0.023069 | -0.036723 | -0.102951 | -0.056684 | -0.007024 | -0.122348 | 0.072403 |
| *Acetoanaerobium* | 0.002774 | -0.037638 | -0.036874 | -0.003166 | 0.01126 | -0.015828 | -0.016738 | -0.009724 | -0.007448 | 0.003249 | -0.036932 | 0.012249 |
| *Acidaminococcus* | -0.006857 | -0.012288 | -0.032386 | -0.007871 | -0.024128 | -0.009823 | -0.046059 | -0.031188 | -0.02706 | -0.046696 | -0.062601 | 0.012178 |
| *Acidovorax* | -0.020186 | -0.047333 | -0.032632 | -0.024201 | -0.004599 | -0.024922 | -0.010951 | -0.004784 | -0.011438 | 0.014724 | -0.029731 | 0.010962 |
| *Acinetobacter* | -0.020313 | 0.012752 | 0.027848 | 0.001046 | -0.007119 | 0.024793 | 0.026797 | 0.009898 | 0.062289 | 0.005229 | 0.001502 | -0.009598 |
| *Actinobaculum* | -0.002443 | 0.011299 | 0.007776 | 0.000437 | 0.011076 | 0.023956 | -0.015138 | -0.020353 | -0.010013 | 0.035821 | -0.030598 | 0.014404 |
| *Actinomadura* | -0.005671 | -0.008132 | 0.018427 | -0.003166 | -0.003581 | 0.004552 | -0.010386 | 0.024932 | -0.025366 | -0.003455 | 0.010324 | -0.023112 |
| *Actinomyces* | 0.000595 | 0.013852 | 0.027846 | 0.015579 | 0.026611 | 0.025811 | 0.021676 | -0.023317 | 0.112594 | 0.059273 | 0.003535 | -0.024615 |
| *Adlercreutzia* | 0.028007 | -0.003056 | 0.012477 | -0.032362 | -0.042917 | -0.005152 | 0.047424 | -0.102091 | 0.110115 | 0.069542 | -0.002254 | -0.016573 |
| *Aeromonas* | 0.020785 | 0.005495 | 0.001344 | 0.01949 | 0.031435 | -0.052153 | -0.00152 | -0.035517 | 0.003544 | 0.019145 | -0.011248 | -0.011151 |
| *Aestuariispira* | -0.026234 | -0.075156 | -0.100871 | -0.064758 | -0.032335 | -0.018662 | -0.041506 | -0.081301 | -0.05292 | -0.000159 | -0.089973 | 0.042806 |
| *Aggregatibacter* | 0.071312 | 0.002266 | -0.012745 | 0.01828 | -0.004684 | 0.007458 | -0.011825 | -0.028945 | -0.031326 | 0.007192 | -0.03357 | 0.014274 |
| *Akkermansia* | -0.015351 | -0.070336 | -0.079994 | -0.063341 | -0.072482 | -0.060799 | -0.017344 | -0.083234 | 0.001841 | 0.056183 | -0.069034 | 0.032083 |
| *Algoriphagus* | -0.005671 | -0.008132 | 0.018427 | -0.003166 | -0.003581 | 0.004552 | -0.010386 | 0.024932 | -0.025366 | -0.003455 | 0.010324 | -0.023112 |
| *Alistipes* | -0.029128 | -0.131803 | -0.152376 | -0.106769 | -0.149796 | -0.115166 | -0.028459 | -0.165987 | -0.041081 | 0.032597 | -0.177245 | 0.114506 |
| *Alkaliphilus* | -0.074357 | -0.023059 | -0.020821 | -0.043953 | -0.054922 | -0.059516 | -0.043101 | -0.048697 | 0.055276 | 0.003828 | -0.069594 | 0.004735 |
| *Alkalitalea* | 0.027302 | 0.004387 | 0.015753 | -0.005257 | -0.006272 | 0.036808 | -0.012559 | 0.018663 | -0.000952 | -0.017815 | 0.033621 | -0.017091 |
| *Allisonella* | -0.095401 | 0.05687 | 0.035573 | 0.02391 | 0.001932 | 0.011177 | 0.027392 | 0.039281 | 0.015834 | -0.038785 | 0.040325 | 0.002725 |
| *Allobaculum* | -0.011229 | 0.001762 | -0.007346 | -0.023431 | 0.004856 | 0.012951 | 0.010993 | -0.007329 | 0.002423 | 0.024149 | -0.017521 | 0.041996 |
| *Alloprevotella* | -0.022776 | 0.014528 | 0.03024 | -0.004355 | 0.048028 | 0.028302 | -0.018058 | -0.010353 | -0.069152 | -0.016637 | 0.021069 | -0.015535 |
| *Altererythrobacter* | -0.0167 | -0.018612 | -0.002394 | -0.013579 | -0.016742 | 0.008735 | -0.003898 | 0.023406 | -0.003324 | 0.011655 | -0.006867 | 0.007092 |
| *Anaerobacter* | -0.008455 | 0.017416 | -0.003223 | 0.012289 | 0.009766 | -0.008958 | -0.021314 | 0.009484 | -0.003865 | -0.027667 | 0.023634 | -0.010394 |
| *Anaerobacterium* | 0.002983 | -0.04599 | -0.053696 | -0.038645 | -0.023761 | 0.008087 | 0.008929 | -0.025328 | 0.010093 | 0.003997 | -0.048447 | 0.017583 |
| *Anaerobiospirillum* | -0.022541 | 0.034141 | 0.03186 | 0.008113 | 0.034463 | 0.019242 | 0.01409 | -0.005131 | -0.004407 | 0.005649 | 0.036642 | 0.012249 |
| *Anaerococcus* | 0.018842 | -0.025099 | -0.036076 | -0.047131 | -0.037896 | 0.022039 | 0.000721 | 0.025649 | 0.007612 | 0.024319 | -0.039875 | 0.022968 |
| *Anaerofilum* | 0.020439 | -0.006527 | 0.016669 | -0.028057 | -0.017854 | 0.00125 | -0.014465 | 0.013255 | -0.018077 | 0.010082 | -0.002272 | 0.00681 |
| *Anaerofustis* | 0.02108 | -0.020172 | -0.009186 | 0.004863 | 0.009513 | -0.029286 | 0.001934 | -0.038051 | 0.025568 | 0.038508 | -0.010293 | -0.033606 |
| *Anaeroglobus* | -0.003933 | 0.003249 | 0.00961 | -0.007471 | 0.016828 | 0.004014 | -0.023194 | 0.024063 | -0.016697 | -0.033624 | 0.023525 | -0.012953 |
| *Anaerophaga* | 0.027301 | -0.001017 | 0.004666 | -0.001109 | -0.000402 | -0.009671 | 0.005277 | -0.026317 | -0.000126 | 0.002589 | -0.012576 | -0.000398 |
| *Anaeroplasma* | 0.012428 | -0.002287 | -0.022112 | -0.013321 | -0.007609 | -0.016211 | -0.015118 | -0.02101 | -0.018596 | 0.001756 | -0.051025 | 0.044586 |
| *Anaerorhabdus* | 0.02281 | 0.00629 | 0.02271 | 0.026925 | 0.026328 | -0.019118 | 0.005959 | 0.0138 | -0.001635 | -0.02634 | 0.018062 | -0.009684 |
| *Anaerostipes* | 0.046411 | 0.0075 | 0.007561 | -0.019371 | -0.047642 | -0.027167 | 0.030517 | -0.046419 | 0.107109 | 0.096596 | 0.060373 | -0.019096 |
| *Anaerotruncus* | -0.027034 | -0.08299 | -0.084853 | -0.08771 | -0.061805 | -0.034116 | -0.068925 | -0.101367 | -0.108123 | -0.010119 | -0.138599 | 0.090498 |
| *Anaerovorax* | 0.043052 | -0.046011 | -0.052594 | -0.050691 | -0.047025 | 0.010864 | 0.033712 | -0.088528 | 0.017717 | 0.082664 | -0.092509 | 0.077919 |
| *Anoxybacillus* | 0.02281 | -0.015125 | -0.01124 | -0.016039 | -0.003581 | 0.016159 | 0.038401 | 0.021725 | 0.031615 | 0.016284 | 0.006228 | -0.0371 |
| *Arachidicoccus* | 0.002774 | -0.037638 | -0.036874 | -0.003166 | 0.01126 | -0.015828 | -0.016738 | -0.009724 | -0.007448 | 0.003249 | -0.036932 | 0.012249 |
| *Arcobacter* | 0.002774 | -0.037638 | -0.036874 | -0.003166 | 0.01126 | -0.015828 | -0.016738 | -0.009724 | -0.007448 | 0.003249 | -0.036932 | 0.012249 |
| *Arenimonas* | -0.005671 | -0.008132 | 0.018427 | -0.003166 | -0.003581 | 0.004552 | -0.010386 | 0.024932 | -0.025366 | -0.003455 | 0.010324 | -0.023112 |
| *Arthrobacter* | -0.005671 | -0.008132 | 0.018427 | -0.003166 | -0.003581 | 0.004552 | -0.010386 | 0.024932 | -0.025366 | -0.003455 | 0.010324 | -0.023112 |
| *Asaccharobacter* | 0.047798 | 0.041702 | 0.041833 | -0.012071 | -0.028586 | -0.00979 | 0.037 | -0.058919 | 0.052716 | 0.05034 | -0.032951 | 0.033537 |
| *Asteroleplasma* | -0.00741 | 0.017484 | 0.025133 | -0.009623 | 0.023948 | 0.018208 | -0.007304 | -0.014897 | -0.005152 | 0.026858 | -0.017235 | -0.011753 |
| *Asticcacaulis* | -0.005671 | -0.008132 | 0.018427 | -0.003166 | -0.003581 | 0.004552 | -0.010386 | 0.024932 | -0.025366 | -0.003455 | 0.010324 | -0.023112 |
| *Atopobium* | 0.029738 | -0.019773 | -0.010368 | 0.011931 | 0.002826 | 0.009892 | -0.004437 | -0.029786 | 0.049555 | 0.017182 | -0.056703 | -0.004446 |
| *Azoarcus* | -0.005671 | -0.008132 | 0.018427 | -0.003166 | -0.003581 | 0.004552 | -0.010386 | 0.024932 | -0.025366 | -0.003455 | 0.010324 | -0.023112 |
| *Azospirillum* | -0.005671 | -0.008132 | 0.018427 | -0.003166 | -0.003581 | 0.004552 | -0.010386 | 0.024932 | -0.025366 | -0.003455 | 0.010324 | -0.023112 |
| *Bacillus* | -0.039173 | -0.009041 | 0.028146 | 0.006557 | -0.022407 | -0.056222 | -0.001599 | -0.015408 | 0.117923 | 0.056186 | 0.015634 | -0.022773 |
| *Bacteroides* | 0.01045 | -0.087193 | -0.100508 | -0.040368 | -0.004557 | 0.004242 | -0.011088 | 0.044913 | -0.132119 | -0.041235 | -0.018432 | 0.046486 |
| *Barnesiella* | -0.045285 | -0.05571 | -0.052807 | -0.104009 | -0.107669 | -0.064185 | -0.048356 | -0.103329 | -0.029368 | -0.027543 | -0.130979 | 0.038882 |
| *Bifidobacterium* | -0.097245 | -0.07021 | -0.106444 | -0.044203 | -0.091078 | -0.105258 | -0.0872 | -0.090622 | 0.003031 | 0.016871 | -0.146516 | 0.09739 |
| *Bilophila* | 0.012171 | -0.041284 | -0.042952 | -0.029957 | -0.058128 | 0.002743 | 0.018543 | -0.022381 | -0.000333 | 0.001111 | -0.027655 | 0.057922 |
| *Blastococcus* | -0.005671 | -0.008132 | 0.018427 | -0.003166 | -0.003581 | 0.004552 | -0.010386 | 0.024932 | -0.025366 | -0.003455 | 0.010324 | -0.023112 |
| *Blautia* | 0.060815 | 0.049876 | 0.064395 | -0.013699 | -0.053018 | -0.004359 | 0.090834 | -0.05179 | 0.200555 | 0.146056 | 0.039133 | -0.025474 |
| *Brevibacillus* | 0.021592 | -0.022731 | -0.02074 | -0.036311 | -0.041343 | -0.010246 | -0.019562 | -0.028889 | 0.043597 | 0.007728 | -0.038144 | -0.024665 |
| *Brevundimonas* | -0.012033 | -0.036939 | -0.023243 | -0.012915 | -0.007164 | -0.00201 | -0.012857 | 0.013504 | -0.007032 | 0.011389 | -0.026933 | 0.012851 |
| *Brochothrix* | 0.012957 | -0.010408 | -0.025488 | 0.025724 | 0.014075 | -0.024994 | -0.01529 | 0.015849 | -0.008897 | 0.033644 | -0.034283 | 0.032444 |
| *Burkholderia* | -0.027778 | -0.03143 | -0.029959 | 0.005733 | 0.005444 | -0.027973 | 0.016863 | -0.016345 | 0.014794 | 0.022223 | -0.025759 | -0.001904 |
| *Buttiauxella* | -0.00571 | 0.006956 | -0.010237 | 0.004096 | -0.031586 | -0.016866 | -0.020605 | -0.023273 | 0.033777 | 0.021339 | -0.010694 | 0.018503 |
| *Butyricicoccus* | -0.010966 | -0.011718 | -0.022524 | -0.036365 | -0.050433 | -0.011822 | -0.008765 | -0.07816 | 0.031251 | 0.041308 | -0.031806 | 0.043081 |
| *Butyricimonas* | -0.037347 | -0.096343 | -0.121216 | -0.091328 | -0.100504 | -0.086955 | -0.06094 | -0.121913 | -0.076444 | -0.003588 | -0.168712 | 0.138515 |
| *Butyrivibrio* | 0.004135 | -0.013363 | -0.057559 | -0.019039 | 0.017691 | -0.027817 | -0.052981 | -0.068942 | -0.118313 | -0.035573 | -0.116031 | 0.075989 |
| *Campylobacter* | 0.007426 | -0.007532 | -0.003097 | -0.025673 | -0.005043 | 0.002929 | -0.056375 | -0.046347 | -0.05006 | -0.004564 | -0.002352 | 0.01972 |
| *Carnobacterium* | -0.00087 | 0.002738 | 0.026384 | -0.037794 | -0.044799 | -0.001244 | 0.00836 | -0.032487 | 0.040136 | 0.024837 | -0.012847 | 0.020581 |
| *Castellaniella* | -0.005671 | -0.008132 | 0.018427 | -0.003166 | -0.003581 | 0.004552 | -0.010386 | 0.024932 | -0.025366 | -0.003455 | 0.010324 | -0.023112 |
| *Catabacter* | 0.013333 | -0.047534 | -0.046807 | -0.021081 | -0.024351 | -0.052399 | 0.008308 | -0.011232 | -0.005743 | 0.005664 | -0.032269 | -0.014687 |
| *Catenibacterium* | -0.056775 | 0.05996 | 0.05628 | 0.02524 | 0.024498 | -0.017532 | -0.005981 | -0.017437 | 0.019036 | -0.008949 | 0.006578 | -0.004519 |
| *Caulobacter* | -0.005671 | -0.008132 | 0.018427 | -0.003166 | -0.003581 | 0.004552 | -0.010386 | 0.024932 | -0.025366 | -0.003455 | 0.010324 | -0.023112 |
| *Cellulosilyticum* | 0.018307 | 0.000852 | 0.020555 | -0.01591 | 0.012216 | 0.038551 | 0.024493 | 0.055434 | -0.001021 | -0.019048 | 0.031525 | -0.053092 |
| *Cellvibrio* | -0.005671 | -0.008132 | 0.018427 | -0.003166 | -0.003581 | 0.004552 | -0.010386 | 0.024932 | -0.025366 | -0.003455 | 0.010324 | -0.023112 |
| *Cerasicoccus* | -0.00741 | 0.017484 | 0.025133 | -0.009623 | 0.023948 | 0.018208 | -0.007304 | -0.014897 | -0.005152 | 0.026858 | -0.017235 | -0.011753 |
| *Cetobacterium* | 0.003346 | 0.009789 | 0.021309 | 0.006508 | 0.024776 | 0.054699 | -0.012191 | 0.069152 | -0.011096 | -0.032318 | 0.007729 | -0.009449 |
| *Chiayiivirga* | -0.005671 | -0.008132 | 0.018427 | -0.003166 | -0.003581 | 0.004552 | -0.010386 | 0.024932 | -0.025366 | -0.003455 | 0.010324 | -0.023112 |
| *Chishuiella* | -0.027176 | -0.02078 | -0.048099 | -0.007526 | -0.031827 | -0.028487 | -0.007195 | 0.00109 | 0.008916 | -0.003351 | -0.004792 | 0.028486 |
| *Chitinophaga* | -0.005671 | -0.008132 | 0.018427 | -0.003166 | -0.003581 | 0.004552 | -0.010386 | 0.024932 | -0.025366 | -0.003455 | 0.010324 | -0.023112 |
| *Christensenella* | -0.04921 | -0.048482 | -0.052121 | -0.040418 | -0.045584 | -0.053476 | -0.022492 | -0.032858 | -0.002881 | -0.015192 | -0.053213 | -0.006945 |
| *Chryseobacterium* | -0.03981 | -0.006447 | 0.006591 | -0.037675 | -0.026763 | -0.010066 | 0.02675 | 0.013189 | 0.02459 | 0.002683 | -0.009879 | -0.031565 |
| *Citrobacter* | -0.005982 | 0.029822 | 0.013238 | -0.012898 | -0.022399 | -0.00078 | 0.017106 | -0.004143 | 0.053692 | 0.002236 | -0.016052 | 0.00185 |
| *Cloacibacillus* | 0.025865 | -0.0368 | -0.012589 | -0.043557 | -0.042278 | 0.009988 | -0.023032 | -0.041991 | -0.034751 | 0.011289 | -0.067667 | 0.013836 |
| *Clostridium_III* | 0.032143 | -0.015441 | -0.002525 | 0.004427 | -0.001237 | -0.025502 | 0.006947 | -0.062851 | 0.020363 | 0.035065 | -0.037148 | 0.012304 |
| *Clostridium_IV* | -0.03102 | -0.071904 | -0.097605 | -0.090561 | -0.10797 | -0.120335 | -0.046803 | -0.188982 | 0.01 | 0.042891 | -0.174136 | 0.076638 |
| *Clostridium_XI* | 0.041386 | 0.039052 | 0.073881 | 0.039384 | 0.027567 | 0.031751 | 0.033493 | -0.01281 | 0.068914 | 0.03945 | 0.055026 | -0.047003 |
| *Clostridium_XVIII* | 0.057902 | 0.035708 | 0.047064 | 0.016685 | -0.003938 | 0.030635 | 0.062527 | 0.0096 | 0.140121 | 0.080311 | 0.07767 | -0.05754 |
| *Clostridium_XlVa* | -0.019379 | -0.068043 | -0.09358 | -0.025467 | 0.018903 | -0.024865 | -0.015939 | 0.002629 | -0.124338 | -0.029548 | -0.036927 | 0.061682 |
| *Clostridium_XlVb* | -0.018392 | -0.061207 | -0.094503 | -0.054133 | -0.034823 | -0.029126 | -0.053186 | 0.003341 | -0.097302 | -0.037228 | -0.064346 | 0.060897 |
| *Clostridium_sensu_stricto* | 0.058359 | 0.078898 | 0.072896 | 0.044867 | 0.031751 | 0.067135 | 0.055526 | -0.00153 | 0.081121 | 0.036746 | 0.049985 | -0.021899 |
| *Collinsella* | -0.117084 | 0.087663 | 0.102414 | -0.019907 | -0.057963 | 0.023685 | -0.007989 | -0.043268 | 0.055968 | 0.003048 | 0.044876 | -0.013026 |
| *Comamonas* | -0.005354 | 0.0091 | -0.012118 | -0.007772 | 0.009701 | -0.004524 | -0.024945 | 0.011575 | -0.042128 | -0.015156 | -0.047165 | 0.033983 |
| *Coprobacillus* | 0.003605 | -0.006119 | 0.010357 | 0.016635 | 0.01361 | -0.015533 | 0.037563 | 0.028758 | 0.04953 | 0.071006 | 0.039892 | -0.029395 |
| *Coprobacter* | -0.03243 | -0.064496 | -0.079699 | -0.08071 | -0.060172 | -0.046892 | -0.049536 | -0.103934 | -0.078902 | 0.031182 | -0.103938 | 0.086721 |
| *Coprococcus* | 0.004582 | 0.027675 | 0.011447 | -0.04148 | -0.055844 | -0.01469 | -0.00227 | -0.133696 | 0.029171 | 0.062166 | -0.068346 | 0.054711 |
| *Coraliomargarita* | 0.020637 | -0.025844 | -0.027034 | -0.03394 | -0.038106 | -0.014256 | -0.030311 | -0.031366 | -0.009952 | -0.023278 | -0.031635 | 0.014174 |
| *Coriobacterium* | 0.003737 | 0.064675 | 0.050731 | 0.027356 | 0.005034 | 0.054096 | 0.016671 | -0.011621 | 0.05327 | -0.002118 | 0.001484 | -0.005053 |
| *Corynebacterium* | -0.002161 | 0.011125 | 0.015441 | 0.006591 | 0.02223 | 0.037974 | 0.025617 | 0.018408 | 0.03046 | 0.022275 | 0.027217 | -0.038444 |
| *Cronobacter* | -0.065106 | -0.025994 | -0.029061 | -0.05359 | -0.068863 | -0.040433 | -0.048644 | -0.062245 | 0.05655 | 0.016894 | -0.058536 | 0.001714 |
| *Curvibacter* | -0.005671 | -0.008132 | 0.018427 | -0.003166 | -0.003581 | 0.004552 | -0.010386 | 0.024932 | -0.025366 | -0.003455 | 0.010324 | -0.023112 |
| *Defluviitalea* | -0.000786 | -0.018517 | -0.026439 | -0.018221 | -0.008001 | -0.019367 | -0.037419 | -0.044424 | -0.046357 | 0.031823 | -0.062148 | 0.028185 |
| *Delftia* | -0.013175 | 0.0304 | 0.022826 | -0.006738 | -0.014773 | -0.015113 | 0.033901 | 0.001347 | 0.07134 | 0.01014 | -0.016861 | -0.019361 |
| *Desulfobaculum* | -0.007172 | -0.000006 | 0.005448 | -0.015367 | 0.000082 | -0.046273 | 0.01688 | -0.010944 | 0.00754 | 0.004551 | 0.0024 | -0.028507 |
| *Desulfobulbus* | 0.034029 | -0.012353 | -0.004262 | 0.010306 | -0.011281 | 0.031946 | 0.039498 | -0.012849 | 0.039498 | 0.033644 | 0.009207 | 0.017898 |
| *Desulfomicrobium* | 0.020454 | -0.014823 | -0.031803 | -0.061629 | -0.067305 | 0.024607 | -0.013428 | -0.042661 | -0.021313 | -0.00121 | -0.008787 | -0.004195 |
| *Desulfovibrio* | 0.025134 | 0.019278 | 0.020022 | 0.019269 | 0.010322 | -0.000294 | 0.002173 | -0.043919 | 0.01799 | 0.037658 | -0.028077 | -0.004539 |
| *Devosia* | -0.005671 | -0.008132 | 0.018427 | -0.003166 | -0.003581 | 0.004552 | -0.010386 | 0.024932 | -0.025366 | -0.003455 | 0.010324 | -0.023112 |
| *Dialister* | -0.117552 | -0.026201 | -0.061889 | -0.084184 | -0.065589 | -0.067408 | -0.080352 | -0.104682 | -0.074166 | -0.038035 | -0.166406 | 0.114688 |
| *Dietzia* | -0.046981 | -0.050608 | -0.046025 | -0.047692 | -0.045316 | 0.009199 | -0.013297 | -0.020136 | -0.014052 | 0.027795 | -0.031838 | 0.042409 |
| *Dokdonella* | -0.005671 | -0.008132 | 0.018427 | -0.003166 | -0.003581 | 0.004552 | -0.010386 | 0.024932 | -0.025366 | -0.003455 | 0.010324 | -0.023112 |
| *Dongia* | -0.005671 | -0.008132 | 0.018427 | -0.003166 | -0.003581 | 0.004552 | -0.010386 | 0.024932 | -0.025366 | -0.003455 | 0.010324 | -0.023112 |
| *Dorea* | 0.00548 | 0.1012 | 0.122191 | 0.011082 | -0.015246 | 0.034973 | 0.061517 | -0.020981 | 0.151975 | 0.102341 | 0.091624 | -0.04633 |
| *Dyadobacter* | -0.005671 | -0.008132 | 0.018427 | -0.003166 | -0.003581 | 0.004552 | -0.010386 | 0.024932 | -0.025366 | -0.003455 | 0.010324 | -0.023112 |
| *Dysgonomonas* | 0.002299 | -0.040984 | -0.047273 | -0.026234 | -0.017038 | -0.027115 | -0.017403 | -0.029406 | 0.00703 | -0.013323 | -0.050839 | 0.009268 |
| *Eggerthella* | 0.085483 | 0.008485 | 0.048141 | -0.017213 | -0.064706 | 0.011378 | 0.042624 | -0.002425 | 0.101684 | 0.081854 | 0.045752 | -0.040421 |
| *Eikenella* | 0.038334 | 0.021561 | 0.00961 | 0.039114 | 0.037423 | 0.021352 | 0.0006 | -0.032939 | 0.01018 | -0.012146 | 0.012807 | -0.028678 |
| *Eisenbergiella* | 0.051576 | -0.028694 | -0.016112 | -0.01868 | -0.009989 | -0.009595 | -0.009463 | -0.042804 | -0.033601 | 0.030299 | -0.047417 | -0.004019 |
| *Elusimicrobium* | -0.017655 | 0.045796 | 0.034352 | 0.045499 | 0.042984 | 0.009078 | 0.030601 | 0.042391 | 0.016501 | -0.036628 | -0.004198 | -0.017138 |
| *Emticicia* | -0.005671 | -0.008132 | 0.018427 | -0.003166 | -0.003581 | 0.004552 | -0.010386 | 0.024932 | -0.025366 | -0.003455 | 0.010324 | -0.023112 |
| *Enhydrobacter* | 0.021836 | 0.019989 | 0.01303 | -0.004576 | -0.025888 | -0.012126 | -0.026691 | -0.045419 | 0.008256 | 0.018789 | -0.0048 | -0.015403 |
| *Ensifer* | -0.002053 | -0.032362 | -0.013027 | -0.004479 | 0.005426 | -0.007968 | -0.019183 | 0.010769 | -0.023216 | -0.000149 | -0.018802 | -0.007696 |
| *Enterobacter* | -0.022224 | -0.057662 | -0.058303 | -0.031285 | -0.040866 | -0.052307 | -0.019437 | -0.071702 | 0.023715 | 0.047655 | -0.029801 | -0.013017 |
| *Enterococcus* | 0.031552 | -0.036027 | -0.035298 | -0.021822 | -0.017303 | -0.006134 | -0.017538 | -0.062024 | 0.058167 | 0.047346 | -0.057943 | -0.008624 |
| *Enterorhabdus* | 0.031574 | 0.012292 | 0.011099 | 0.029952 | 0.009261 | 0.011963 | -0.01329 | -0.021825 | -0.004944 | 0.033625 | -0.019335 | 0.01774 |
| *Escherichia* | 0.002751 | 0.053505 | 0.061427 | 0.041382 | 0.063599 | 0.03319 | 0.017667 | 0.059277 | 0.059666 | 0.033389 | 0.073775 | -0.042859 |
| *Ethanoligenens* | 0.020848 | 0.003081 | 0.012391 | -0.009353 | 0.021751 | -0.005894 | 0.023475 | 0.005768 | 0.031957 | 0.04189 | 0.027381 | -0.043607 |
| *Eubacterium* | 0.018983 | -0.054519 | -0.044763 | -0.067798 | -0.0537 | -0.008342 | 0.014606 | -0.102211 | 0.048089 | 0.016361 | -0.07686 | 0.002776 |
| *Exiguobacterium* | -0.024065 | -0.031908 | -0.017115 | -0.008101 | -0.01621 | -0.011044 | -0.031705 | 0.008529 | -0.025369 | -0.020129 | -0.015216 | -0.030928 |
| *Ezakiella* | 0.023954 | -0.011266 | 0.00137 | -0.020556 | -0.004083 | 0.055123 | 0.013883 | 0.023864 | 0.014259 | -0.013532 | -0.025183 | 0.030592 |
| *Faecalibacterium* | 0.018356 | -0.03518 | -0.063809 | -0.057827 | -0.080913 | -0.065342 | -0.004436 | -0.184605 | 0.020569 | 0.068819 | -0.093999 | 0.047992 |
| *Faecalicoccus* | 0.039534 | -0.001035 | 0.019868 | -0.009623 | 0.023948 | 0.012456 | 0.032442 | 0.0138 | -0.014421 | 0.005649 | 0.010469 | -0.039417 |
| *Faecalitalea* | 0.022239 | -0.001479 | -0.008417 | -0.01021 | -0.004982 | -0.030853 | -0.012961 | -0.043415 | -0.031077 | 0.016612 | -0.026123 | 0.045803 |
| *Falsiporphyromonas* | -0.003536 | -0.023781 | -0.018805 | -0.017526 | -0.010672 | 0.002921 | 0.023747 | -0.001802 | 0.010124 | 0.026504 | 0.006979 | -0.006962 |
| *Fibrobacter* | -0.005671 | -0.008132 | 0.018427 | -0.003166 | -0.003581 | 0.004552 | -0.010386 | 0.024932 | -0.025366 | -0.003455 | 0.010324 | -0.023112 |
| *Fictibacillus* | -0.005671 | -0.008132 | 0.018427 | -0.003166 | -0.003581 | 0.004552 | -0.010386 | 0.024932 | -0.025366 | -0.003455 | 0.010324 | -0.023112 |
| *Finegoldia* | -0.034154 | -0.038428 | -0.038056 | -0.047364 | -0.041176 | -0.017605 | 0.006903 | 0.001841 | 0.017031 | 0.014686 | -0.037005 | 0.015384 |
| *Flavisolibacter* | -0.005671 | -0.008132 | 0.018427 | -0.003166 | -0.003581 | 0.004552 | -0.010386 | 0.024932 | -0.025366 | -0.003455 | 0.010324 | -0.023112 |
| *Flavobacterium* | 0.01836 | -0.001035 | 0.005766 | -0.009623 | 0.014075 | 0.03207 | -0.003517 | -0.027477 | 0.030559 | -0.027064 | -0.013511 | 0.013408 |
| *Flavonifractor* | 0.045433 | -0.072542 | -0.058354 | -0.036564 | -0.04185 | -0.044441 | 0.005246 | -0.025471 | -0.042732 | 0.059502 | -0.023922 | 0.034351 |
| *Fontibacillus* | 0.036078 | -0.008132 | 0.02271 | 0.012355 | 0.019436 | 0.032277 | -0.021559 | 0.014731 | -0.000952 | -0.02634 | 0.011048 | -0.034182 |
| *Fusicatenibacter* | -0.020383 | 0.019622 | -0.002851 | 0.005401 | -0.031727 | -0.023167 | 0.022607 | -0.076474 | 0.097908 | 0.07057 | -0.040335 | 0.02592 |
| *Fusobacterium* | -0.02383 | 0.056176 | 0.10454 | 0.014434 | 0.051442 | 0.089785 | 0.053811 | 0.184272 | -0.020349 | -0.063938 | 0.187842 | -0.090536 |
| *Gaiella* | 0.003924 | -0.023022 | -0.01758 | -0.023337 | -0.041079 | -0.02219 | 0.008315 | -0.010383 | 0.012393 | 0.013011 | 0.00355 | -0.022474 |
| *Gallicola* | -0.020743 | -0.051924 | -0.05299 | -0.023357 | -0.009705 | -0.000515 | -0.00968 | -0.003507 | 0.005203 | 0.012912 | -0.047353 | 0.032834 |
| *Gardnerella* | 0.045165 | -0.035551 | -0.021045 | -0.040121 | -0.03511 | -0.005632 | -0.005505 | -0.016262 | -0.008856 | 0.04147 | -0.04284 | 0.047248 |
| *Gemella* | 0.05691 | 0.007636 | 0.011196 | 0.024727 | -0.002082 | 0.05749 | 0.044145 | -0.003242 | 0.099963 | 0.070226 | 0.032241 | -0.001926 |
| *Gemmatimonas* | -0.005671 | -0.008132 | 0.018427 | -0.003166 | -0.003581 | 0.004552 | -0.010386 | 0.024932 | -0.025366 | -0.003455 | 0.010324 | -0.023112 |
| *Gemmiger* | -0.053166 | -0.013656 | -0.039402 | -0.053297 | -0.08794 | -0.070308 | -0.019947 | -0.180615 | 0.088434 | 0.049047 | -0.115682 | 0.060201 |
| *Geobacillus* | -0.020086 | 0.000087 | -0.008363 | 0.012399 | 0.003309 | 0.029107 | -0.029712 | -0.025165 | 0.041157 | 0.010079 | -0.00998 | 0.001173 |
| *Gordonibacter* | 0.035212 | 0.013318 | 0.026337 | -0.01586 | -0.054555 | 0.013097 | 0.025131 | -0.009846 | 0.062018 | 0.0409 | 0.005708 | 0.002375 |
| *Granulicatella* | 0.087809 | 0.011017 | 0.015818 | 0.012025 | -0.000804 | 0.022106 | 0.023726 | -0.026723 | 0.081512 | 0.091145 | 0.011667 | -0.001514 |
| *Haemophilus* | -0.015055 | -0.04326 | -0.06691 | 0.016015 | -0.007968 | -0.057713 | -0.061661 | -0.150876 | -0.010929 | 0.031202 | -0.095078 | 0.054486 |
| *Hafnia* | -0.012359 | -0.012694 | -0.008634 | 0.000508 | 0.008339 | 0.019777 | -0.004593 | -0.003397 | 0.002221 | -0.019591 | 0.020583 | 0.033147 |
| *Haloferula* | -0.005671 | -0.008132 | 0.018427 | -0.003166 | -0.003581 | 0.004552 | -0.010386 | 0.024932 | -0.025366 | -0.003455 | 0.010324 | -0.023112 |
| *Helicobacter* | -0.050514 | -0.024632 | -0.011783 | -0.037301 | -0.030771 | 0.017319 | 0.011945 | -0.031104 | 0.00711 | 0.030982 | -0.007277 | 0.003604 |
| *Hespellia* | -0.006538 | -0.018247 | -0.021557 | 0.009439 | 0.007436 | -0.017378 | 0.01393 | 0.000347 | -0.011912 | 0.039886 | -0.045918 | 0.016431 |
| *Holdemanella* | -0.038931 | 0.024993 | 0.017993 | 0.003443 | 0.003532 | -0.025292 | -0.017456 | -0.050711 | -0.001437 | -0.005134 | -0.074251 | 0.019649 |
| *Holdemania* | 0.01157 | -0.019606 | 0.01729 | -0.012397 | -0.004629 | -0.007369 | 0.008898 | 0.008166 | 0.004963 | 0.046578 | 0.014997 | -0.010225 |
| *Howardella* | -0.02178 | -0.001893 | -0.023817 | -0.020244 | 0.016176 | -0.024083 | -0.007744 | -0.029768 | -0.006411 | -0.019361 | -0.043226 | 0.035916 |
| *Hungatella* | -0.020919 | -0.012993 | -0.022266 | -0.019923 | -0.001884 | -0.010752 | 0.004731 | -0.01197 | 0.02252 | -0.028861 | -0.019463 | 0.001791 |
| *Hydrogenoanaerobacterium* | 0.008181 | -0.038292 | -0.013035 | -0.017321 | -0.004918 | -0.052603 | 0.00815 | -0.053394 | 0.023373 | 0.025124 | -0.046167 | 0.023326 |
| *Hydrogenophaga* | -0.000429 | -0.016447 | 0.010027 | -0.028545 | -0.029223 | 0.015142 | -0.001266 | -0.004706 | -0.011354 | -0.007623 | 0.010275 | -0.04052 |
| *Imperialibacter* | -0.005671 | -0.008132 | 0.018427 | -0.003166 | -0.003581 | 0.004552 | -0.010386 | 0.024932 | -0.025366 | -0.003455 | 0.010324 | -0.023112 |
| *Inhella* | -0.005671 | -0.008132 | 0.018427 | -0.003166 | -0.003581 | 0.004552 | -0.010386 | 0.024932 | -0.025366 | -0.003455 | 0.010324 | -0.023112 |
| *Insolitispirillum* | -0.017946 | 0.017484 | 0.016567 | -0.009623 | -0.015669 | -0.027187 | 0.028015 | 0.000952 | 0.006414 | 0.009725 | 0.005297 | 0.013408 |
| *Intestinibacter* | 0.013729 | -0.020612 | -0.01567 | -0.027302 | -0.03758 | -0.031744 | 0.038863 | -0.085026 | 0.120814 | 0.087764 | -0.041723 | 0.019806 |
| *Intestinimonas* | -0.018664 | -0.057225 | -0.076358 | -0.105792 | -0.112003 | -0.054499 | -0.020453 | -0.177135 | 0.007921 | 0.056537 | -0.13846 | 0.085905 |
| *Isobaculum* | 0.033097 | -0.005773 | 0.019659 | 0.037417 | 0.026328 | 0.033043 | -0.003807 | -0.037015 | -0.025366 | 0.025719 | -0.034325 | 0.034492 |
| *Klebsiella* | 0.046382 | 0.002265 | 0.000775 | 0.053571 | 0.020254 | 0.053358 | 0.011347 | -0.010838 | 0.039308 | -0.016679 | -0.044043 | -0.028413 |
| *Kluyvera* | -0.00387 | -0.017472 | -0.023895 | -0.003105 | 0.003305 | 0.012872 | 0.004314 | 0.025153 | 0.039637 | 0.004863 | 0.012763 | -0.032224 |
| *Kofleria* | -0.005671 | -0.008132 | 0.018427 | -0.003166 | -0.003581 | 0.004552 | -0.010386 | 0.024932 | -0.025366 | -0.003455 | 0.010324 | -0.023112 |
| *Kribbella* | -0.005671 | -0.008132 | 0.018427 | -0.003166 | -0.003581 | 0.004552 | -0.010386 | 0.024932 | -0.025366 | -0.003455 | 0.010324 | -0.023112 |
| *Lachnospira* | 0.000476 | -0.035217 | -0.032947 | 0.005733 | -0.026598 | -0.015828 | -0.014442 | -0.037367 | -0.032608 | 0.037596 | -0.039415 | 0.011132 |
| *Lacibacter* | -0.005671 | -0.008132 | 0.018427 | -0.003166 | -0.003581 | 0.004552 | -0.010386 | 0.024932 | -0.025366 | -0.003455 | 0.010324 | -0.023112 |
| *Lacibacterium* | -0.005671 | -0.008132 | 0.018427 | -0.003166 | -0.003581 | 0.004552 | -0.010386 | 0.024932 | -0.025366 | -0.003455 | 0.010324 | -0.023112 |
| *Lactobacillus* | -0.013561 | 0.032006 | 0.027288 | 0.007823 | -0.027049 | -0.000347 | -0.000053 | -0.025984 | 0.040393 | 0.005803 | -0.066635 | 0.049403 |
| *Lactococcus* | 0.022916 | -0.005604 | 0.019445 | 0.02516 | -0.006349 | -0.017611 | 0.028632 | -0.016918 | 0.145095 | 0.072023 | 0.039717 | -0.061348 |
| *Lactonifactor* | 0.046994 | 0.019715 | 0.014697 | 0.017253 | -0.010128 | -0.030047 | -0.014508 | 0.010171 | -0.00569 | 0.021005 | -0.031109 | -0.025816 |
| *Lactovum* | 0.010018 | 0.004387 | -0.004262 | 0.018667 | 0.03171 | -0.029773 | 0.012166 | -0.016345 | 0.029132 | 0.016284 | -0.015828 | -0.0395 |
| *Leclercia* | -0.004028 | 0.00706 | 0.005728 | 0.006359 | 0.003386 | 0.005629 | -0.021698 | 0.01778 | 0.014231 | -0.023042 | 0.003064 | 0.024332 |
| *Leptotrichia* | -0.015089 | -0.037638 | -0.03491 | 0.005733 | -0.015669 | -0.039043 | -0.023711 | -0.00091 | -0.023194 | 0.000683 | -0.035608 | 0.013408 |
| *Leuconostoc* | -0.021619 | -0.007118 | -0.022292 | -0.015843 | -0.01254 | -0.012506 | -0.020836 | -0.037824 | 0.00052 | -0.004701 | -0.026045 | 0.010788 |
| *Limnobacter* | -0.005671 | -0.008132 | 0.018427 | -0.003166 | -0.003581 | 0.004552 | -0.010386 | 0.024932 | -0.025366 | -0.003455 | 0.010324 | -0.023112 |
| *Luteibacter* | -0.032083 | 0.007449 | 0.002361 | -0.022682 | -0.036616 | -0.027187 | 0.024559 | -0.004552 | 0.034201 | -0.006145 | 0.009952 | -0.031472 |
| *Luteimonas* | -0.005671 | -0.008132 | 0.018427 | -0.003166 | -0.003581 | 0.004552 | -0.010386 | 0.024932 | -0.025366 | -0.003455 | 0.010324 | -0.023112 |
| *Luteolibacter* | -0.005671 | -0.008132 | 0.018427 | -0.003166 | -0.003581 | 0.004552 | -0.010386 | 0.024932 | -0.025366 | -0.003455 | 0.010324 | -0.023112 |
| *Lysobacter* | -0.005671 | -0.008132 | 0.018427 | -0.003166 | -0.003581 | 0.004552 | -0.010386 | 0.024932 | -0.025366 | -0.003455 | 0.010324 | -0.023112 |
| *Macellibacteroides* | 0.026041 | -0.032362 | -0.039004 | -0.026257 | -0.014832 | 0.01193 | -0.000515 | -0.003214 | -0.010537 | -0.005332 | -0.017162 | 0.021325 |
| *Macrococcus* | 0.033097 | -0.013636 | 0.00961 | 0.005733 | 0.016828 | 0.031946 | -0.034387 | -0.008442 | -0.031677 | -0.01078 | -0.019097 | 0.038134 |
| *Marinospirillum* | 0.00741 | -0.027499 | 0.011491 | 0.003042 | 0.03171 | 0.00569 | 0.016863 | 0.034201 | -0.013449 | 0.004407 | -0.004097 | -0.031472 |
| *Massilia* | -0.0167 | -0.011503 | 0.024176 | -0.003119 | -0.015279 | -0.015793 | -0.006677 | 0.020101 | -0.006586 | 0.006843 | 0.026189 | -0.04052 |
| *Megamonas* | -0.082397 | 0.063698 | 0.049261 | 0.009118 | 0.05148 | 0.045679 | -0.024262 | 0.100896 | -0.124995 | -0.143975 | 0.079591 | -0.013701 |
| *Megasphaera* | -0.070833 | 0.0349 | -0.000526 | 0.007261 | -0.011066 | -0.01854 | -0.038069 | 0.017982 | -0.056551 | -0.084295 | -0.014929 | 0.025535 |
| *Melissococcus* | 0.036078 | 0.038466 | 0.037731 | 0.039011 | 0.038416 | -0.007221 | -0.023194 | 0.002007 | -0.005152 | -0.036727 | -0.03087 | 0.006104 |
| *Mesorhizobium* | -0.005671 | -0.008132 | 0.018427 | -0.003166 | -0.003581 | 0.004552 | -0.010386 | 0.024932 | -0.025366 | -0.003455 | 0.010324 | -0.023112 |
| *Methanobrevibacter* | -0.009133 | -0.00905 | -0.019897 | 0.01111 | 0.00867 | 0.003825 | 0.011712 | -0.019165 | 0.022486 | 0.031461 | -0.054195 | 0.056395 |
| *Methanosphaera* | 0.012957 | -0.030748 | -0.025488 | -0.019516 | 0.005444 | 0.005276 | -0.007862 | -0.025263 | 0.025449 | -0.001117 | -0.035939 | 0.03892 |
| *Methylobacillus* | -0.005671 | -0.008132 | 0.018427 | -0.003166 | -0.003581 | 0.004552 | -0.010386 | 0.024932 | -0.025366 | -0.003455 | 0.010324 | -0.023112 |
| *Methylobacterium* | 0.012957 | -0.010408 | -0.025488 | 0.025724 | 0.014075 | -0.024994 | -0.01529 | 0.015849 | -0.008897 | 0.033644 | -0.034283 | 0.032444 |
| *Methylotenera* | -0.005671 | -0.008132 | 0.018427 | -0.003166 | -0.003581 | 0.004552 | -0.010386 | 0.024932 | -0.025366 | -0.003455 | 0.010324 | -0.023112 |
| *Methyloversatilis* | -0.005671 | -0.008132 | 0.018427 | -0.003166 | -0.003581 | 0.004552 | -0.010386 | 0.024932 | -0.025366 | -0.003455 | 0.010324 | -0.023112 |
| *Methylovorus* | -0.005671 | -0.008132 | 0.018427 | -0.003166 | -0.003581 | 0.004552 | -0.010386 | 0.024932 | -0.025366 | -0.003455 | 0.010324 | -0.023112 |
| *Mitsuokella* | -0.035412 | 0.004206 | -0.001742 | -0.00231 | 0.016675 | -0.009014 | -0.013112 | 0.016868 | -0.034121 | -0.059659 | -0.033087 | -0.012852 |
| *Mogibacterium* | 0.024609 | -0.04166 | -0.010864 | -0.038644 | -0.053779 | -0.016514 | -0.016912 | -0.091182 | 0.047933 | 0.080204 | -0.042125 | 0.042979 |
| *Morganella* | 0.01196 | -0.006749 | 0.015786 | -0.006718 | 0.000363 | 0.007313 | -0.040316 | 0.003631 | -0.010974 | -0.023016 | 0.008684 | 0.001959 |
| *Moryella* | -0.020367 | 0.004387 | -0.015878 | 0.003042 | 0.029288 | 0.013594 | 0.000207 | 0.016925 | -0.007945 | -0.032962 | -0.019345 | 0.017132 |
| *Mucispirillum* | -0.001222 | 0.011364 | 0.004352 | -0.007623 | -0.011675 | 0.012093 | -0.014465 | 0.010314 | -0.029696 | 0.015294 | 0.025222 | 0.017813 |
| *Murdochiella* | -0.004361 | -0.020059 | -0.01605 | -0.028709 | -0.016999 | 0.013811 | -0.025676 | -0.029325 | -0.013136 | 0.028956 | -0.062322 | 0.048565 |
| *Murimonas* | 0.044538 | 0.013757 | 0.013275 | 0.044898 | 0.050878 | -0.000608 | 0.002607 | 0.030822 | -0.012397 | -0.015693 | 0.016254 | -0.015087 |
| *Mycoplasma* | 0.035105 | -0.029816 | -0.013141 | -0.032222 | -0.024962 | 0.007221 | -0.038525 | 0.016325 | -0.039001 | 0.025057 | -0.035525 | 0.022636 |
| *Negativicoccus* | 0.002162 | -0.046391 | -0.036194 | -0.043899 | -0.035311 | 0.015773 | -0.025113 | 0.014916 | -0.017142 | 0.028337 | -0.046362 | 0.040168 |
| *Neisseria* | 0.033854 | 0.012278 | 0.015977 | -0.004102 | -0.019935 | 0.062957 | -0.007739 | -0.01449 | 0.002509 | -0.030669 | 0.015423 | -0.034994 |
| *Novosphingobium* | -0.005671 | -0.008132 | 0.018427 | -0.003166 | -0.003581 | 0.004552 | -0.010386 | 0.024932 | -0.025366 | -0.003455 | 0.010324 | -0.023112 |
| *Oceanobacillus* | -0.002221 | -0.037453 | -0.033316 | -0.025639 | -0.026901 | -0.044815 | -0.013282 | 0.006164 | 0.020236 | -0.024905 | -0.022436 | -0.035258 |
| *Ochrobactrum* | -0.01996 | 0.039758 | 0.0444 | -0.011692 | -0.017883 | 0.009456 | 0.032285 | 0.008996 | 0.047719 | -0.008155 | -0.014387 | -0.031865 |
| *Odoribacter* | -0.087454 | -0.146635 | -0.152855 | -0.109888 | -0.115335 | -0.10394 | -0.074112 | -0.175555 | -0.090915 | 0.012222 | -0.182143 | 0.129589 |
| *Olsenella* | -0.022083 | -0.018637 | 0.01213 | -0.031416 | -0.036803 | 0.00021 | -0.031929 | -0.048543 | -0.00092 | 0.015112 | -0.00152 | 0.022032 |
| *Opitutus* | -0.005671 | -0.008132 | 0.018427 | -0.003166 | -0.003581 | 0.004552 | -0.010386 | 0.024932 | -0.025366 | -0.003455 | 0.010324 | -0.023112 |
| *Oribacterium* | 0.013705 | -0.002221 | -0.00186 | -0.015857 | 0.034141 | 0.027891 | -0.005494 | 0.02139 | -0.021698 | -0.027597 | 0.033483 | -0.050363 |
| *Oscillibacter* | -0.037121 | -0.158599 | -0.191038 | -0.126699 | -0.149677 | -0.118053 | -0.083493 | -0.23545 | -0.081984 | 0.046492 | -0.242857 | 0.137975 |
| *Oxalobacter* | 0.020423 | -0.020707 | -0.023974 | -0.060064 | -0.042654 | -0.057395 | -0.010598 | -0.083772 | -0.005412 | 0.050858 | -0.06002 | 0.046193 |
| *Paenibacillus* | -0.094784 | -0.048535 | -0.041857 | -0.023837 | -0.050857 | -0.059558 | -0.032382 | -0.056892 | 0.087484 | 0.013523 | -0.100194 | 0.004392 |
| *Pantoea* | -0.009749 | 0.030251 | 0.00961 | -0.013866 | -0.003581 | 0.01649 | 0.009269 | 0.000952 | 0.025594 | -0.012146 | 0.020256 | -0.001904 |
| *Papillibacter* | 0.054693 | 0.008979 | 0.001769 | 0.033512 | 0.014978 | 0.016206 | 0.008017 | -0.025718 | -0.010614 | 0.012267 | 0.017609 | -0.015979 |
| *Parabacteroides* | -0.038887 | -0.152842 | -0.178225 | -0.069651 | -0.070692 | -0.078072 | -0.028285 | -0.072324 | -0.089212 | 0.007349 | -0.140075 | 0.097983 |
| *Paracoccus* | -0.017086 | 0.024417 | 0.026888 | 0.007315 | -0.0006 | 0.009993 | -0.00705 | 0.009243 | 0.017525 | 0.00265 | 0.027235 | -0.001021 |
| *Paraeggerthella* | -0.007603 | -0.00361 | -0.021177 | -0.04137 | -0.046937 | 0.000735 | 0.010613 | -0.016291 | 0.021105 | 0.022294 | 0.001298 | 0.011086 |
| *Paraprevotella* | -0.013488 | -0.070801 | -0.062338 | -0.04116 | -0.030003 | -0.041825 | -0.066357 | -0.056504 | -0.105945 | -0.024306 | -0.083038 | 0.022816 |
| *Parasegetibacter* | -0.005671 | -0.008132 | 0.018427 | -0.003166 | -0.003581 | 0.004552 | -0.010386 | 0.024932 | -0.025366 | -0.003455 | 0.010324 | -0.023112 |
| *Parasporobacterium* | 0.051311 | -0.047119 | -0.052194 | -0.034025 | -0.042204 | 0.006042 | 0.02022 | -0.038932 | -0.007843 | 0.034454 | -0.05567 | 0.046356 |
| *Parasutterella* | -0.010081 | -0.08173 | -0.10442 | -0.029538 | -0.025833 | -0.029251 | -0.058797 | -0.020949 | -0.136887 | -0.038752 | -0.072928 | 0.033695 |
| *Parvibacter* | -0.017649 | -0.011263 | -0.005285 | 0.000863 | -0.01983 | 0.025578 | 0.03539 | -0.010872 | 0.047089 | 0.02909 | 0.008881 | 0.000437 |
| *Parvimonas* | -0.00576 | 0.014756 | -0.001282 | -0.004891 | 0.009173 | 0.025292 | -0.000211 | -0.037411 | 0.036405 | 0.020195 | -0.019003 | 0.026586 |
| *Pediococcus* | -0.010707 | 0.01537 | 0.019807 | 0.036808 | 0.044149 | -0.005033 | -0.013209 | 0.017098 | 0.00098 | -0.04974 | -0.027012 | 0.010589 |
| *Pedobacter* | -0.005671 | -0.008132 | 0.018427 | -0.003166 | -0.003581 | 0.004552 | -0.010386 | 0.024932 | -0.025366 | -0.003455 | 0.010324 | -0.023112 |
| *Peptococcus* | 0.020746 | -0.025432 | -0.01546 | -0.032582 | -0.027131 | -0.000763 | -0.013194 | -0.069774 | -0.011904 | 0.017881 | -0.053646 | 0.021754 |
| *Peptoniphilus* | -0.024826 | -0.033061 | -0.034293 | -0.009239 | -0.019814 | -0.005666 | -0.053815 | -0.03568 | -0.034273 | 0.027843 | -0.076 | 0.044097 |
| *Peptostreptococcus* | 0.03971 | -0.051746 | -0.078131 | -0.048163 | -0.039545 | 0.011928 | -0.019513 | -0.02213 | -0.020211 | 0.023627 | -0.031159 | 0.008656 |
| *Peredibacter* | -0.005671 | -0.008132 | 0.018427 | -0.003166 | -0.003581 | 0.004552 | -0.010386 | 0.024932 | -0.025366 | -0.003455 | 0.010324 | -0.023112 |
| *Phascolarctobacterium* | 0.01897 | -0.077813 | -0.086773 | 0.017634 | 0.004937 | -0.017955 | 0.008962 | -0.00414 | -0.018982 | 0.04749 | -0.008565 | 0.033837 |
| *Phenylobacterium* | -0.005671 | -0.008132 | 0.018427 | -0.003166 | -0.003581 | 0.004552 | -0.010386 | 0.024932 | -0.025366 | -0.003455 | 0.010324 | -0.023112 |
| *Phocaeicola* | -0.001863 | -0.018188 | -0.014269 | -0.016039 | 0.021547 | 0.022987 | -0.013842 | 0.027456 | -0.03027 | 0.003249 | 0.034325 | -0.029713 |
| *Plesiomonas* | -0.003551 | 0.021939 | 0.020432 | 0.004593 | -0.003916 | -0.012541 | 0.007583 | 0.005334 | 0.016956 | 0.034975 | 0.054119 | -0.016586 |
| *Pontibacter* | -0.005671 | -0.008132 | 0.018427 | -0.003166 | -0.003581 | 0.004552 | -0.010386 | 0.024932 | -0.025366 | -0.003455 | 0.010324 | -0.023112 |
| *Porphyromonas* | 0.019443 | -0.016548 | -0.001206 | -0.024076 | -0.018892 | 0.018743 | -0.010912 | -0.028487 | 0.003265 | 0.016398 | -0.025608 | 0.011721 |
| *Povalibacter* | -0.005671 | -0.008132 | 0.018427 | -0.003166 | -0.003581 | 0.004552 | -0.010386 | 0.024932 | -0.025366 | -0.003455 | 0.010324 | -0.023112 |
| *Prevotella* | -0.046597 | 0.022507 | 0.008075 | -0.001661 | 0.019599 | 0.007918 | -0.063556 | -0.035441 | -0.096599 | -0.089757 | -0.059188 | 0.032314 |
| *Propionispira* | -0.018129 | -0.0037 | 0.009175 | 0.022538 | 0.029026 | -0.000783 | -0.011774 | 0.00688 | -0.027285 | 0.020934 | -0.010565 | 0.027344 |
| *Proteiniphilum* | -0.009749 | -0.004697 | -0.01124 | -0.033133 | -0.026598 | 0.004552 | -0.006104 | 0.03867 | 0.013366 | -0.023278 | 0.035897 | -0.016056 |
| *Proteus* | -0.000651 | 0.027162 | 0.007934 | -0.006334 | -0.001622 | 0.0256 | 0.009281 | -0.015216 | 0.015646 | 0.038413 | 0.00017 | -0.015205 |
| *Pseudobutyrivibrio* | 0.024184 | -0.024591 | -0.024635 | 0.00577 | 0.033757 | 0.021632 | 0.018461 | -0.013314 | 0.037331 | -0.003287 | 0.012124 | -0.037582 |
| *Pseudocitrobacter* | 0.004822 | -0.014458 | -0.025152 | -0.001503 | -0.024349 | 0.010967 | 0.026619 | -0.003168 | 0.061885 | 0.010246 | 0.013454 | 0.002331 |
| *Pseudoflavonifractor* | 0.029279 | -0.016846 | -0.014677 | -0.036109 | -0.026297 | 0.068336 | 0.009272 | -0.019772 | -0.017071 | 0.006813 | -0.032795 | 0.022409 |
| *Pseudomonas* | 0.026843 | -0.004356 | -0.000797 | -0.021528 | -0.023956 | 0.030932 | 0.025351 | 0.00951 | 0.096947 | 0.031621 | 0.0158 | -0.026725 |
| *Pseudoscardovia* | -0.003933 | -0.010408 | -0.006957 | 0.016535 | 0.019436 | 0.001221 | 0.036435 | 0.032877 | 0.014442 | 0.006704 | 0.006414 | -0.025016 |
| *Pseudoxanthomonas* | -0.005671 | -0.008132 | 0.018427 | -0.003166 | -0.003581 | 0.004552 | -0.010386 | 0.024932 | -0.025366 | -0.003455 | 0.010324 | -0.023112 |
| *Psychrobacter* | 0.034584 | -0.0071 | -0.021307 | -0.020831 | -0.033854 | 0.051339 | 0.030736 | 0.018682 | -0.001884 | 0.000495 | -0.0102 | 0.018678 |
| *Pyramidobacter* | -0.017637 | -0.01607 | -0.025243 | 0.014415 | 0.003111 | -0.016648 | 0.005795 | -0.026652 | -0.00629 | 0.02463 | -0.053158 | 0.054351 |
| *Ramlibacter* | -0.005671 | -0.008132 | 0.018427 | -0.003166 | -0.003581 | 0.004552 | -0.010386 | 0.024932 | -0.025366 | -0.003455 | 0.010324 | -0.023112 |
| *Raoultella* | -0.007255 | -0.008882 | -0.032151 | -0.001744 | -0.016944 | -0.008665 | 0.010117 | -0.03229 | 0.0628 | 0.029638 | -0.014856 | 0.015625 |
| *Rheinheimera* | -0.005671 | -0.008132 | 0.018427 | -0.003166 | -0.003581 | 0.004552 | -0.010386 | 0.024932 | -0.025366 | -0.003455 | 0.010324 | -0.023112 |
| *Rhizobium* | -0.005671 | -0.008132 | 0.018427 | -0.003166 | -0.003581 | 0.004552 | -0.010386 | 0.024932 | -0.025366 | -0.003455 | 0.010324 | -0.023112 |
| *Rhodanobacter* | -0.005671 | -0.008132 | 0.018427 | -0.003166 | -0.003581 | 0.004552 | -0.010386 | 0.024932 | -0.025366 | -0.003455 | 0.010324 | -0.023112 |
| *Rhodobacter* | 0.002774 | -0.037638 | -0.036874 | -0.003166 | 0.01126 | -0.015828 | -0.016738 | -0.009724 | -0.007448 | 0.003249 | -0.036932 | 0.012249 |
| *Rhodococcus* | -0.017946 | -0.018188 | -0.021832 | -0.016039 | -0.020098 | 0.0078 | 0.004883 | 0.008152 | 0.02069 | 0.019947 | -0.020049 | 0.033168 |
| *Rhodocytophaga* | -0.005671 | -0.008132 | 0.018427 | -0.003166 | -0.003581 | 0.004552 | -0.010386 | 0.024932 | -0.025366 | -0.003455 | 0.010324 | -0.023112 |
| *Robinsoniella* | 0.029661 | 0.007449 | 0.025133 | -0.016039 | 0.008259 | -0.023877 | 0.035794 | 0.018663 | 0.038215 | -0.001117 | 0.016614 | -0.016056 |
| *Romboutsia* | -0.008387 | 0.083628 | 0.09904 | 0.050758 | 0.026475 | 0.019977 | 0.062995 | 0.021434 | 0.146956 | 0.046762 | 0.10525 | -0.081535 |
| *Roseburia* | -0.031062 | -0.055215 | -0.072198 | -0.023404 | -0.034944 | -0.048824 | -0.020719 | -0.092043 | -0.050282 | 0.003623 | -0.076463 | 0.029851 |
| *Rothia* | 0.053715 | 0.013476 | 0.021724 | -0.028879 | -0.023718 | 0.044803 | -0.007051 | -0.03869 | 0.025646 | 0.015886 | 0.025128 | -0.02839 |
| *Ruminococcus* | 0.050861 | -0.068707 | -0.078174 | -0.048687 | -0.069364 | -0.058928 | 0.01153 | -0.144489 | 0.04346 | 0.058578 | -0.103699 | 0.013658 |
| *Ruminococcus2* | 0.009486 | 0.075477 | 0.083011 | -0.002533 | -0.058583 | -0.003194 | 0.070422 | -0.03166 | 0.145401 | 0.08501 | 0.078155 | -0.039948 |
| *Saccharofermentans* | 0.002162 | -0.046391 | -0.036194 | -0.043899 | -0.035311 | 0.015773 | -0.025113 | 0.014916 | -0.017142 | 0.028337 | -0.046362 | 0.040168 |
| *Salinimicrobium* | -0.005671 | -0.008132 | 0.018427 | -0.003166 | -0.003581 | 0.004552 | -0.010386 | 0.024932 | -0.025366 | -0.003455 | 0.010324 | -0.023112 |
| *Salmonella* | 0.015653 | 0.008848 | 0.000784 | -0.026577 | -0.002811 | 0.019447 | -0.011242 | -0.02191 | 0.005853 | 0.003726 | -0.01162 | 0.015027 |
| *Sandaracinus* | -0.005671 | -0.008132 | 0.018427 | -0.003166 | -0.003581 | 0.004552 | -0.010386 | 0.024932 | -0.025366 | -0.003455 | 0.010324 | -0.023112 |
| *Selenomonas* | -0.015089 | -0.034182 | -0.031589 | -0.011734 | -0.024962 | -0.03745 | -0.038153 | -0.034967 | -0.035835 | -0.016429 | -0.033497 | 0.006104 |
| *Senegalimassilia* | -0.027171 | 0.03937 | 0.05667 | 0.014159 | 0.020716 | -0.002894 | -0.008071 | -0.040718 | 0.022468 | 0.00055 | -0.013003 | -0.013006 |
| *Shannon* | -0.007529 | -0.022159 | -0.017404 | -0.056399 | -0.096542 | -0.054063 | 0.013645 | -0.131026 | 0.146779 | 0.103958 | -0.041796 | 0.000398 |
| *Slackia* | 0.02065 | 0.043568 | 0.048445 | 0.014124 | -0.008742 | -0.000322 | 0.021825 | -0.04948 | 0.052883 | 0.040785 | 0.004358 | -0.002666 |
| *Sneathia* | 0.035105 | -0.029816 | -0.013141 | -0.032222 | -0.024962 | 0.007221 | -0.038525 | 0.016325 | -0.039001 | 0.025057 | -0.035525 | 0.022636 |
| *Solobacterium* | -0.010105 | -0.023097 | -0.014378 | -0.008055 | -0.025042 | 0.005861 | 0.01232 | -0.013926 | -0.00157 | 0.047004 | -0.012233 | 0.006952 |
| *Sphingobium* | -0.017946 | -0.018188 | -0.021832 | -0.016039 | -0.020098 | 0.0078 | 0.004883 | 0.008152 | 0.02069 | 0.019947 | -0.020049 | 0.033168 |
| *Sphingomonas* | -0.026085 | -0.03449 | -0.020895 | 0.015542 | -0.001978 | -0.020563 | -0.008864 | -0.014819 | 0.011052 | 0.034368 | -0.019675 | -0.005042 |
| *Sphingopyxis* | -0.005671 | -0.008132 | 0.018427 | -0.003166 | -0.003581 | 0.004552 | -0.010386 | 0.024932 | -0.025366 | -0.003455 | 0.010324 | -0.023112 |
| *Sporobacter* | -0.014821 | -0.071753 | -0.072536 | -0.07192 | -0.060973 | -0.043146 | -0.050155 | -0.103455 | -0.012686 | 0.012609 | -0.106174 | 0.061541 |
| *Staphylococcus* | -0.002518 | 0.01584 | 0.002107 | -0.013647 | -0.018098 | 0.009223 | 0.002451 | -0.002787 | 0.041636 | -0.009345 | 0.006811 | -0.008312 |
| *Stenotrophomonas* | -0.009697 | 0.030393 | 0.015537 | -0.018983 | -0.04185 | -0.006659 | 0.016225 | -0.00378 | 0.059413 | 0.00109 | -0.021415 | -0.010232 |
| *Steroidobacter* | -0.005671 | -0.008132 | 0.018427 | -0.003166 | -0.003581 | 0.004552 | -0.010386 | 0.024932 | -0.025366 | -0.003455 | 0.010324 | -0.023112 |
| *Streptococcus* | -0.019961 | 0.025707 | 0.022043 | -0.012899 | -0.039019 | -0.017367 | 0.02061 | -0.084181 | 0.157319 | 0.125873 | -0.012536 | 0.03231 |
| *Streptomyces* | -0.005671 | -0.008132 | 0.018427 | -0.003166 | -0.003581 | 0.004552 | -0.010386 | 0.024932 | -0.025366 | -0.003455 | 0.010324 | -0.023112 |
| *Subdoligranulum* | 0.005071 | -0.033065 | -0.027034 | -0.031043 | -0.029785 | -0.035608 | -0.010738 | -0.027477 | 0.001738 | -0.004821 | -0.033456 | 0.006104 |
| *Succinatimonas* | 0.014859 | 0.012312 | 0.0049 | -0.041694 | -0.01023 | 0.00914 | 0.004137 | -0.027379 | -0.039442 | 0.008217 | 0.020319 | -0.010318 |
| *Succinivibrio* | 0.000506 | 0.007248 | 0.022448 | 0.010238 | 0.035162 | 0.058704 | -0.035364 | -0.001647 | -0.05115 | -0.018165 | -0.002214 | -0.013142 |
| *Sutterella* | -0.080193 | -0.006846 | -0.030718 | -0.00961 | 0.001529 | -0.00021 | -0.018845 | 0.012289 | -0.078797 | -0.043104 | -0.044065 | 0.068526 |
| *Tepidimicrobium* | 0.002113 | -0.046395 | -0.036212 | -0.043897 | -0.035311 | 0.015778 | -0.025082 | 0.014908 | -0.017102 | 0.028329 | -0.046358 | 0.040177 |
| *Terrimonas* | -0.005671 | -0.008132 | 0.018427 | -0.003166 | -0.003581 | 0.004552 | -0.010386 | 0.024932 | -0.025366 | -0.003455 | 0.010324 | -0.023112 |
| *Terrisporobacter* | 0.021443 | 0.008863 | 0.039874 | 0.006993 | -0.019224 | -0.020775 | 0.049159 | 0.004674 | 0.122624 | 0.054182 | 0.023474 | -0.023978 |
| *Thalassobaculum* | -0.005671 | -0.008132 | 0.018427 | -0.003166 | -0.003581 | 0.004552 | -0.010386 | 0.024932 | -0.025366 | -0.003455 | 0.010324 | -0.023112 |
| *Thauera* | 0.002774 | -0.037638 | -0.036874 | -0.003166 | 0.01126 | -0.015828 | -0.016738 | -0.009724 | -0.007448 | 0.003249 | -0.036932 | 0.012249 |
| *Thermoleophilum* | -0.005671 | -0.008132 | 0.018427 | -0.003166 | -0.003581 | 0.004552 | -0.010386 | 0.024932 | -0.025366 | -0.003455 | 0.010324 | -0.023112 |
| *Thermomonas* | -0.005671 | -0.008132 | 0.018427 | -0.003166 | -0.003581 | 0.004552 | -0.010386 | 0.024932 | -0.025366 | -0.003455 | 0.010324 | -0.023112 |
| *Thiobacillus* | -0.009749 | -0.004697 | -0.008148 | 0.018667 | 0.03794 | -0.033767 | -0.011628 | -0.008442 | 0.01018 | -0.006145 | 0.032339 | -0.029713 |
| *Thiopseudomonas* | -0.001863 | 0.014215 | 0.00773 | -0.013866 | 0.005444 | -0.002586 | 0.024766 | 0.033808 | 0.005897 | -0.029858 | 0.006414 | -0.028678 |
| *Tissierella* | 0.018088 | -0.029048 | -0.029107 | -0.003119 | 0.017918 | -0.003975 | -0.021001 | -0.014707 | 0.002583 | -0.0203 | -0.051073 | 0.00036 |
| *Treponema* | 0.027302 | 0.004387 | 0.015753 | -0.005257 | -0.006272 | 0.036808 | -0.012559 | 0.018663 | -0.000952 | -0.017815 | 0.033621 | -0.017091 |
| *Turicibacter* | -0.000849 | 0.00014 | 0.005308 | 0.00464 | 0.007368 | -0.021032 | 0.048767 | -0.015414 | 0.072354 | 0.028202 | -0.024365 | -0.010293 |
| *Ureaplasma* | -0.025128 | 0.037928 | 0.039005 | -0.027256 | -0.024962 | -0.007738 | -0.026794 | -0.013842 | -0.003973 | -0.035755 | 0.024911 | -0.020091 |
| *Vampirovibrio* | 0.000717 | -0.004398 | -0.030971 | -0.007797 | -0.01541 | -0.02498 | -0.00423 | -0.006548 | -0.001821 | -0.009682 | -0.085744 | 0.038956 |
| *Veillonella* | 0.02404 | -0.026212 | -0.040783 | -0.00658 | 0.016589 | -0.020433 | -0.053459 | -0.090983 | -0.022794 | 0.055412 | -0.046321 | -0.004704 |
| *Vibrio* | 0.005071 | 0.026051 | -0.004262 | 0.014425 | 0.034463 | 0.001738 | -0.006104 | -0.027477 | 0.014794 | 0.002028 | -0.017524 | 0.009166 |
| *Victivallis* | -0.021967 | -0.084207 | -0.082802 | -0.078835 | -0.077803 | -0.07498 | -0.039397 | -0.077231 | -0.012429 | -0.009789 | -0.135596 | 0.054444 |
| *Viridibacillus* | -0.008023 | -0.033264 | -0.024214 | -0.027025 | -0.021391 | -0.03917 | -0.008439 | -0.002955 | 0.021673 | -0.010311 | -0.015437 | -0.015438 |
| *Vogesella* | -0.005671 | -0.008132 | 0.018427 | -0.003166 | -0.003581 | 0.004552 | -0.010386 | 0.024932 | -0.025366 | -0.003455 | 0.010324 | -0.023112 |
| *Weissella* | 0.014499 | 0.053996 | 0.054815 | -0.006363 | -0.00747 | -0.005485 | -0.002501 | -0.007458 | 0.021175 | 0.020239 | -0.014303 | 0.031525 |
| *Wohlfahrtiimonas* | 0.029661 | -0.017215 | -0.01124 | 0.02262 | 0.005444 | 0.039374 | 0.001593 | -0.024063 | 0.029401 | -0.008525 | -0.003786 | -0.00658 |

***BMI:** Body mass index; **WL:** Waistline; **SBP:** Systolic blood pressure; **DBP:** Diastolic blood pressure; **GLU:** Blood glucose; **TC:** Total cholesterol; **TG:** Triglyceride; **LDL:** Low density lipoprotein; **HDL:** High density lipoprotein; **UA:** Uric acid; **eGFR:** epidermal growth factor receptor.

**Supplementary scripts**

################Script for PCoa#################################################

data<-read.table(".//Genus_PCoA.xls",head=T,check.names=F)

Group <-c(rep("ClusterI",991),rep("ClusterII",522),rep("ClusterIII",401))

label <- factor(Group)

k<- length(legend)

colors<-c("#3CB371","#FF69B4","#1E90FF")

PCOA <- pcoa(data, correction="none", rn=NULL) #####PCoA

result <-PCOA$values[,"Relative_eig"]

pro1 = as.numeric(sprintf("%.3f",result[1]))*100

pro2 = as.numeric(sprintf("%.3f",result[2]))*100

x = PCOA$vectors

sample_names = rownames(x)

pc = as.data.frame(PCOA$vectors)

pc$names = sample_names

legend_title = ""

group = Group

pc$group = group

xlab=paste("PCoA1(",pro1,"%)",sep="")

ylab=paste("PCoA2(",pro2,"%)",sep="")

pdf(".//Genus_PCoa.pdf",width=8,height=8)

ggplot(pc,aes(Axis.1,Axis.2)) +geom_point(size=2,aes(color=group,shape=group))+labs(x=xlab,y=ylab,title="PCoA on bray_curtis diversity distance",color=legend_title,shape=label) +scale_color_manual(values=colors)+stat_ellipse(aes(fill=group),geom="polygon",level=0.95,alpha=0.2)+scale_fill_manual(values= colors)+geom_hline(yintercept=0,linetype=4,color="black")+geom_vline(xintercept=0,linetype=4,color="black")+theme_bw()+theme(panel.grid.major= element_line(color = "white"),panel.grid.minor =element_line(color= "white"),legend.title = element_blank())

####################### Script for PERMANOVA###########################################

library(vegan)

phe<-read.table("Table_phenotype.BMI.eGFR.Group.Area.filter.xls",head=T)

Cluster<-read.table("RDP20180719_profling_Genus.older.xls",head=T,check.names=F)

Cluster1<-t(Cluster)

t<-adonis(Cluster1~Gender+Age+BMI+WL+SBP+DBP+GLU+TC+TG+LDL+HDL+UA+eGFR+Area,data=phe,na.rm=TRUE)

write.table(t,file="adonis_all.txt")

####################### Script for Random forest construction#####################################################

library(randomForest)

library(RColorBrewer)

col<-brewer.pal(9,'Paired')

data<-read.table("./RDP20180327_Genus_train.xls",header=T)

pdf("./H-O.pdf",width=12,height=12)

result<-rfcv(data[2:193],data$class,scale="log",cv.fold=5,step=0.5)

plot(result$n.var, result$error.cv, log="x", type="o", lwd=2)

ntree_fit<-randomForest(class~.,data=data,mtry=50,ntree=1000,importance=TRUE)

importance<-importance(x=ntree_fit)

write.table(importance, file = "./Data10-fold.importance.txt", append = T, quote = F, sep = "\t", row.names = T)

print<-cbind(result$n.var,result$error.cv)

write.table(print, file = "./Data1.values.txt", append = T, quote = F, sep = "\t", row.names = T)

for (i in 2:10) {

rf<-randomForest(class~.,data=data,mtry=50,ntree=1000,importance=TRUE)

result<-rfcv(data[2:193],data$class,scale="log",cv.fold=5,step=0.5)

#lines(result$n.var, result$error.cv, log="x", type="o", lwd=2,col=col[i])

plot(result$n.var, result$error.cv, log="x", type="o", lwd=2)

importance<-importance(x=rf)

write.table(importance, file = "./Data10-fold.importance.txt", append = T, quote = F, sep = "\t", row.names = T)

print<-cbind(result$n.var,result$error.cv)

write.table(print, file = "./Data1.values.txt", append = T, quote = F, sep = "\t", row.names = T)

}

library(caret)

library(pROC) #ROC

library(e1071)

library(randomForest) #RF

library(adabag) #adaboosting

library(dplyr)

library(DMwR)

library(RColorBrewer)

perf <- function(t, n=2){

tcase <- t[1,1]/sum(t[1,])

tcon <- t[2,2]/sum(t[2,])

acc <- (t[1,1]+t[2,2])/sum(t)

r <- tcase ### recall

p <- t[1,1]/sum(t[,1]) ### presicion

Fscore <- 2*(p*r)/(p+r)

lst <- list(acc = acc, tcase = tcase, tcon = tcon, Fscore = Fscore)

return(lst)

}

#################Test1

file<-list.files("/whg3/Product/33.Obesity_16S/Analysis20180607/1.Mark/1.H-O/Gini_10x/AUC",pattern ="RDP20180615_Genus_Validation",full.names = TRUE,recursive = TRUE)

Peak<-read.table("./Peak.txt",header=F,check.names=F)

for(i in 1:10){

file <- file[i]

set.seed(110)

bg <-read.table(file = file, header = T)

bg$class <- factor(bg$class)

#set.seed(100)

folds <- createFolds(y=bg$class, k = 5)

aucs <- c()

rocs <- data.frame()

for (i in 1:5) {

#foldtest <-read.table(file = "./RDP20180511_H_O_Genus_older.xls", header = T)

foldtest <-bg[folds[[i]],]

# print(nrow(foldtest))

#bg1<-SMOTE(class~ .,bg, perc.over =50,perc.under=210)

foldtrain <- bg[-folds[[i]],]

rf <- randomForest(class~ .,mtry=Peak[i,2],ntree=800,data = foldtrain,importance = TRUE,proximity = TRUE)

importance<-importance(x=rf)

rf_pred <- predict(rf,foldtest)

write.table(importance, file = "./Data5-fold.Validation.txt", append = T, quote = F, sep = "\t", row.names = T)

rf_prob <- predict(rf,foldtest, type = "prob")

t_rf <- table(foldtest$class, rf_pred)

rf_roc <- roc(foldtest$class, rf_prob[,2])

# plot(rf_roc, print.auc=TRUE, auc.polygon=TRUE,max.auc.polygon=TRUE)

# print(length(rf_roc$sensitivities))

a <- confusionMatrix(rf_pred, foldtest$class)

overall <- cbind(t(data.frame(a$overall)),t(data.frame(a$byClass)), roc=rf_roc$auc)

write.table(overall, file = "./Data1.Validation.confusion.txt", append = T, quote = F, sep = "\t", row.names = F)

aucs <- c(aucs, rf_roc$auc)

temp <- data.frame(index=i,sens=rf_roc$sensitivities, spec = rf_roc$specificities)

temp2 <- data.frame(rp = rownames(temp),temp)

rocs <- rbind(rocs, temp2)

}

print(file)

print(aucs)

meanroc <- group_by(rocs,rp) %>% summarise_all(funs(mean))

meanroc <- arrange(meanroc,desc(sens), spec)

rocs_all <- data.frame()

tmp<-data.frame(index=1,sens=meanroc$sens, spec = meanroc$spec)

tmp2<-data.frame(rp = rownames(tmp),tmp)

rocs_all <-rbind(rocs_all,tmp2)

aucs_mean <- c()

aucs_mean<-c(aucs_mean,round(mean(aucs/1),3))

}

dev.off()
